# Progress and Prospect of Ion Imprinting Technology in Targeted Extraction of Lithium

**DOI:** 10.3390/polym16060833

**Published:** 2024-03-18

**Authors:** Keke Zhi, Jinwang Duan, Jiarui Zhang, Lianting Huang, Lianghui Guo, Lulu Wang

**Affiliations:** 1 College of Engineering, China University of Petroleum-Beijing at Karamay, Karamay 834000, China; zhikeke@cupk.edu.cn (K.Z.); duanjw1997@163.com (J.D.); jrzhang@st.cupk.edu.cn (J.Z.); 2023015732@st.cupk.edu.cn (L.H.); 2 State Key Laboratory of Heavy Oil Processing-Karamay Branch, Karamay 834000, China; 3 College of Chemistry, Xinjiang University, Urumqi 830017, China; 4 State Key Laboratory of Chemistry and Utilization of Carbon Based Energy Resources, Urumqi 830017, China

**Keywords:** ion imprinting technology, lithium ion-imprinted polymers, targeted lithium extraction

## Abstract

Ion Imprinting Technology (IIT) is an innovative technique that produces Ion-Imprinted polymers (IIPs) capable of selectively extracting ions. IIPs exhibit strong specificity, excellent stability, and high practicality. Due to their superior characteristics, the application of IIPs for lithium resource extraction has garnered significant attention. This paper discusses the following aspects based on existing conventional processes for lithium extraction and the latest research progress in lithium IIPs: (1) a detailed exposition of existing lithium extraction processes, including comparisons and summaries; (2) classification, comparison, and summarization of the latest lithium IIPs based on different material types and methods; (3) summarization of the applications of various lithium IIPs, along with a brief description of future directions in the development of lithium IIP applications. Finally, the prospects for targeted recovery of lithium resources using lithium IIPs are presented.

## 1. Introduction

Lithium (Li) is a metallic element located in the second period and group IA, making it one of the alkali metals. It holds the distinction of being the lightest metal and solid element under standard conditions. Thanks to its unique physical and chemical properties [1], lithium finds wide-ranging applications in various industries including batteries, ceramics, glass, lubricants, refrigeration fluids, the nuclear industry, and the photoelectrical industry [2]. It is often referred to as the “industrial monosodium glutamate” [3]; with its exceptionally high standard oxidation potential among all elements, lithium is particularly well suited for use in batteries and energy storage applications, earning it the titles of “21st century energy metal” and “white oil”. The market demand for lithium salts, such as lithium carbonate, lithium hydroxide, and lithium chloride, which serve as core raw materials in the lithium industry, has experienced significant growth in recent years due to the rapid development of new energy vehicles, chemical energy storage, and other industries.

The lithium content in the Earth’s crust is approximately 0.0065%, ranking twenty-seventh in abundance. Global lithium resources are diverse, including pegmatite, clay, lithium zeolite, oil and gas field brine, geothermal brine, and saline brine types. Among these, pegmatite and saline brine are dominant [4,5,6]. However, the distribution of these resources is uneven, with concentrations in regions such as the South American lithium triangle (near Argentina, Bolivia, and Chile), Australia, China, the United States, the Democratic Republic of Congo (DRC), and Canada [7]. Lithium deposits are globally distributed, primarily in western Australia, the Tibetan Plateau in China, Congo (DRC), and other regions. Salt lake brine-type lithium deposits are predominantly found in the South American lithium triangle, with secondary occurrences in the Tibetan Plateau in China [8] and the west coast of the United States. Currently, global lithium resource development is primarily influenced by factors such as the uneven distribution of resources, the natural environment, government policies, development conditions, and technology. The main sources of global lithium resources are ores and salt lake brine. However, due to technological limitations and cost considerations, lithium extraction from ores remains the predominant method, with other types of lithium extraction being less common.

China possesses diverse lithium resources, encompassing ore and salt lake type. Analogous to the global lithium distribution, a substantial proportion of China’s lithium resources are derived from salt lake deposits. Geographically, lithium resources are predominantly situated in provinces and regions such as Qinghai, Tibet, Sichuan, Jiangxi, Xinjiang, Hunan, and others. Specifically, Sichuan and Xinjiang predominantly feature lithium pyroxene, while Jiangxi and Hunan showcase mainly lithium mica. Tibet and Qinghai, on the other hand, are characterized by brine-type saline lake lithium resources [9]. China’s saline brine lithium deposits generally exhibit low lithium content and a high magnesium–lithium ratio. These deposits are predominantly situated in the plateau regions of Qinghai and Tibet, known for their delicate ecological environments. Additionally, inadequate infrastructure, including transportation and electricity, poses challenges for establishing large-scale chemical industries, thereby complicating lithium extraction from these salt lakes.

**Figure 1:**
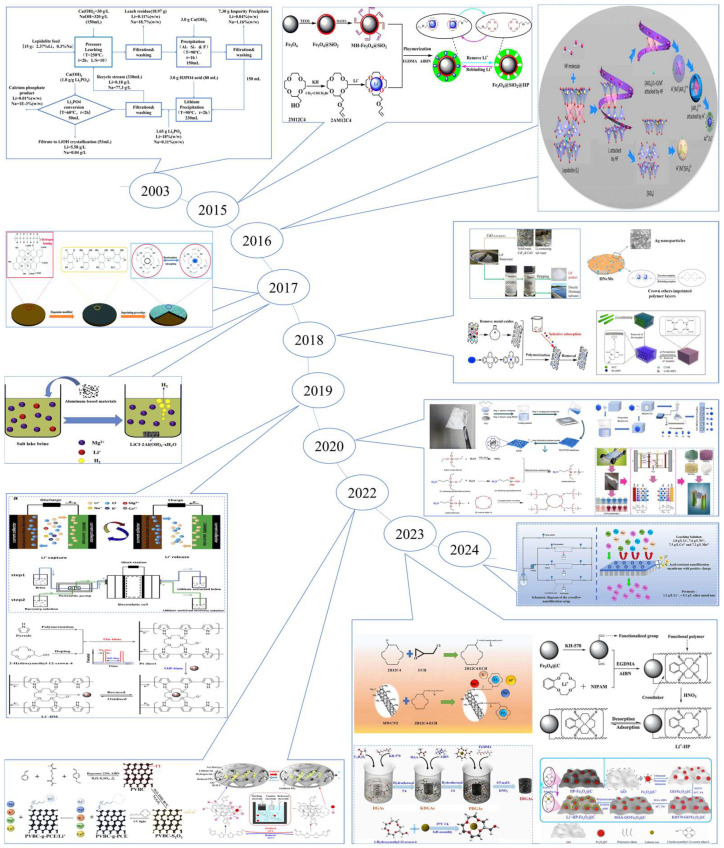
Latest progress in the application of lithium extraction resources [31,32,35,36,37,38,39,40,41,42,43,44,45,46,47]. Reproduced with permissions from Ref. [31], *Elsevier*, 2023; Ref. [32], *Elsevier*, 2021; Ref. [35], *Elsevier*, 2018. Ref. [36], *Elsevier*, 2023; Ref. [37], *Elsevier*, 2022; Ref. [38], *ACS Publications*, 2015; Ref. [39], *Elsevier*, 2018; Ref. [40], *Elsevier*, 2018; Ref. [41], *RCS Publishing*, 2017; Ref. [42], *Elsevier*, 2020; Ref. [43], *Elsevier*, 2023; Ref. [44], *Elsevier*, 2022; Ref. [45], *Elsevier*, 2024; Ref. [46], *Elsevier*, 2020; Ref. [47], *Elsevier*, 2022.

The extraction of lithium resources can be categorized based on their source: from ore and from brine found in salt lakes. In recent years, the lithium extraction process has been extensively studied, and its development process is shown in Figure 1. Common methods for extracting lithium from ores include roasting [10,11] (such as lime, sulfate, and chlorination roasting), acid dissolution [12,13,14] (including sulfuric acid digestion and direct acid leaching), and alkaline leaching [15] (such as high-pressure alkaline leaching). While these methods offer broad applicability and straightforward reaction conditions, they suffer from drawbacks including high energy consumption, large solvent usage, limited processing capacity, challenging purification processes, and significant environmental impact. The commonly used methods for lithium extraction from salt lakes include chemical precipitation [16,17,18], extraction [19,20], electrochemical methods [21], membrane processes [22,23], and adsorption [24]. However, these methods often exhibit low selectivity for lithium ions during adsorption. Leveraging the specificity and selectivity of ion imprinting, materials produced through ion-imprinting technology (IIT) can achieve targeted ion adsorption. Thus, preparing lithium ion-imprinted polymers using this technology presents an ideal approach for adsorbing lithium ions in complex water samples.

Ion-imprinted materials are initially created by uniting monomer molecules with specific recognition functions and template ions. This union occurs through chemical forces, typically involving electrostatic, ligand, and chelating forces resulting in the formation of a prepolymer. By choosing an appropriate cross-linking agent, the polymerization reaction takes place under the influence of the initiator. Afterward, through a specific elution process, the template ions are removed. This results in the formation of three-dimensional cavities within the ion-imprinted polymer. These cavities exhibit complete complementary to the template ions in spatial structure and matching––complementary in terms of charge, number of ligands, ionic size, and geometrical shape. Additionally, these cavities contain functional groups that are specifically bound to the template ions. The three-dimensional cavities exhibit specific recombination with the template ions, demonstrating excellent recognition specificity. Consequently, they have found extensive application in lithium extraction through adsorption studies.

The conventional methods for preparing lithium ion-imprinted materials include suspension polymerization [25], precipitation polymerization [26], and intrinsic polymerization [27]. These methods primarily involve two mechanisms: the sol-gel method [28] and free-radical polymerization [29]. While these conventional approaches are straightforward, they suffer from drawbacks such as limited adsorption capacity, dispersed imprinted sites, low yield, challenging recovery, and elution. However, the integration of new technologies including column separation [30], magnetic separation [31,32], membrane separation [33], and electrochemical methods [34] with conventional approaches for producing various lithium ion-imprinted materials (such as adsorption column materials, magnetic materials, and membrane materials) partially addresses certain issues and charts a course for the advancement of lithium ion-imprinting technology.

This paper analyzes and summarizes current lithium resource extraction processes. (1) It introduces the extraction process from ore and salt lake brine; (2) It describes the traditional methods of extracting lithium from both ore and salt lake brine, highlighting their characteristics and shortcomings; (3) It explains the principle behind preparing lithium ion-imprinted materials; (4) It focuses on the research progress of combining new technologies with the conventional method to prepare various types of lithium ion-imprinted compounds for extracting lithium from salt lake brine. It compares existing lithium ion-imprinted material technologies highlighting their respective advantages and disadvantages, while also summarizing current challenges associated with lithium ion-imprinted materials. Additionally, it discusses the application conditions and potential future improvement directions for different lithium ion-imprinted materials in the extraction process; (5) It discusses the potential of these materials in directing extraction of lithium ions for recycling lithium resources.

## 2. The Current Lithium Extraction Process and Its Advantages and Disadvantages

The current production processes are primarily categorized based on the form of lithium resources: extraction from ores or extraction from salt lakes. Below, we will describe these two processes, provide a brief comparison, and discuss their respective advantages and disadvantages.

### 2.1. Lithium Ore Lithium Extraction Process

The present lithium ore available for exploitation primarily falls into two categories: hard rock lithium ore and clay lithium ore. Hard rock lithium ore consists mainly of lithium pyroxene and lithium mica, while clay lithium ore primarily contains lithium chlorite [4,5,6]. The main extraction methods for these ores can be broadly categorized as follows.

#### 2.1.1. Acid Process

The acid method involves reacting crushed and ground lithium ore with an acid solution under specific conditions. In this reaction, the H^+^ ions in the acid solution replace the valent metal ions in the ore, converting them into soluble salts. Subsequently, through leaching and other processes, the metal ions transition from the solid phase to the liquid phase. Further purification is then carried out to obtain a lithium salt purification solution, which is finally precipitated to separate lithium carbonate.

##### Sulfuric Acid Roasting Method

**Figure 2:**
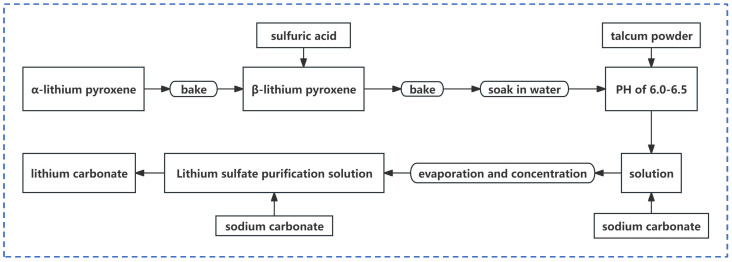
Diagram of the lithium extraction process by sulfuric acid roasting of lithium pyroxene [13]. Reproduced with permission from *Taylor & Francis*, 2020.

Sulfuric acid is commonly utilized in the industry due to its ability to handle various grades of lithium ore, coupled with a relatively straightforward process. However, its high corrosiveness necessitates more stringent equipment requirements. Additionally, during ore roasting with sulfuric acid, significant acid mist is generated, leading to environmental pollution. To address this issue, an acid washing tower is employed to treat the tail gas, resulting in increased slag production. Figure 2 illustrates the lithium extraction process via sulfuric acid roasting of lithium pyroxene [13]

##### Fluorochemical Method

**Figure 3:**
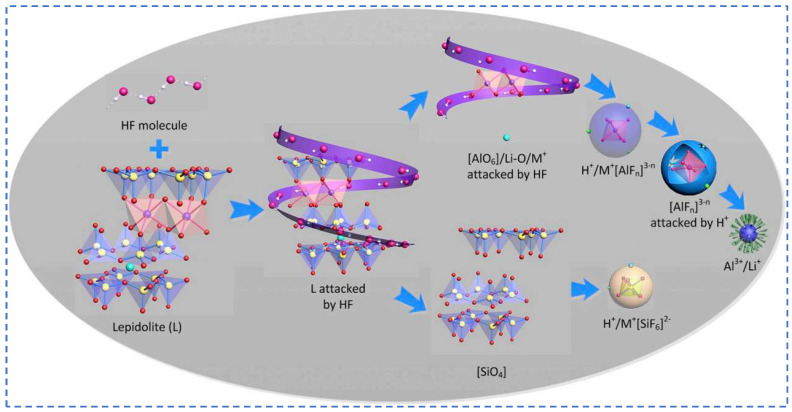
Specific process of dissolution of lithium mica in solution containing HF [52]. Reproduced with permission from *Elsevier*, 2019.

HF can break down silicate minerals at low temperatures, producing silicon tetrafluoride with strong covalency and releasing significant heat. If lithium mica contains 2~3% mass fraction of elemental fluorine, HF can dismantle the Si-O bond in lithium mica ore, enabling the extraction of valuable metals like lithium from the mineral [48,49,50,51]. The process of dissolving lithium mica in an HF-containing solution is illustrated in Figure 3. The fluorine chemical method, an enhanced acid-based approach, offers benefits such as low leaching temperature, brief reaction duration, and high extraction efficiency. This method is not confined solely to lithium extraction; it can also retrieve other rare and precious metals. However, its drawbacks are evident: fluorine, a component of the process, poses environmental and health risks.

Hui Guo et al. [52] employed a combination of HF and H_2_SO_4_ acids to extract lithium ions from lithium mica. Their findings indicated that under conditions where ore/HF/H_2_SO_4_ = 1:2:3.5 (g/mL/mL) at 85 °C, approximately 98% of Li^+^ and 90% of Rb^+^ and Cs^+^ could be efficiently converted into the sulfate form within the leach solution. The addition of H_2_SO_4_ expedited ore dissolution while simultaneously reducing HF consumption. Furthermore, the HF/F^−^ remnants in the post-leach solution, along with the generated AlF_3_ and K_2_SiF_6_, could be reintroduced into the reaction system as a source of HF, thus facilitating further recycling and enhancing economic efficiency.

#### 2.1.2. Alkali Process

The lithium mica alkali process involves a refined technique. Its core revolves around the reaction between lithium mica and concentrated lye, which results in the extraction of Li^+^ into solution. This process can be further classified based on the types of lye used, namely the alkaline solution method and the mixed alkaline solution method.

##### Alkali Method

This method primarily involves the reaction between lithium ore and pure NaOH solution to extract lithium ions. Subsequently, the leaching solution undergoes a series of purification steps, culminating in the addition of sodium carbonate to precipitate lithium carbonate. This method necessitates high-pressure equipment due to its rigorous requirements. Additionally, during the precipitation of lithium ions, there is a phenomenon of co-precipitation with other cations.

##### Mixed Alkali Method

The mixed alkali solubilization method involves utilizing two or more alkali reagents as leaching agents. This approach aims to diminish leaching impurities and enhance the leaching rate of lithium ions.

**Figure 4:**
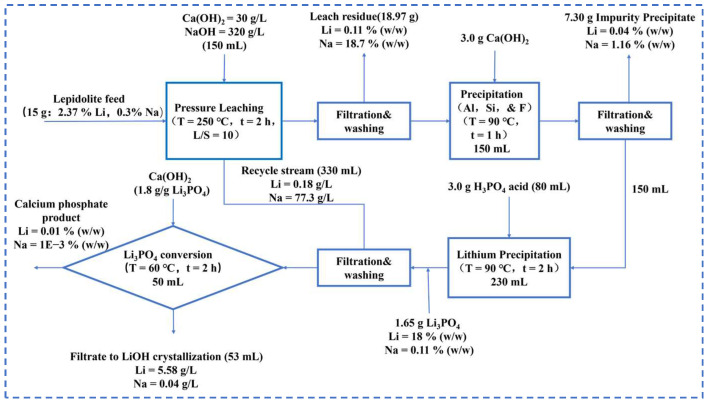
Flow chart of LiOH recovery [15]. Reproduced with permission from *Elsevier*, 2021.

James Mulwanda et al. [15] conducted a study on the pressurized leaching of lithium and other valuable metals from lithium mica using NaOH and Ca(OH)_2_. They found that under optimum conditions (250 °C, 320 g·L^−1^ NaOH, 10 mL·g^−1^ liquid to solid ratio, 300 rpm/min stirring speed, and 0.3 g·g^−1^ lime addition) with a 2 h reaction time, the leaching rates were as follows: Li^+^ 94%, K^+^ 98%, Rb^2+^ 96%, and Cs^+^ 90%. The addition of lime significantly increased the lithium extraction rate. Due to the low solubility of Li_3_PO_4_, the researchers precipitated the lithium ions into Li_3_PO_4_ by adding phosphoric acid, which does not contain metal ions. They then converted the Li_3_PO_4_ to LiOH with Ca (OH)_2_, recovering 83% of the lithium in the form of LiOH. Figure 4 below shows the flow chart for this study.

#### 2.1.3. Salt Process

This process involves roasting lithium-containing ore with salt at a specific temperature. This action destroys the internal crystal structure of the ore, facilitating the exchange of Li^+^ with alkali metal ions in the salt. Consequently, a soluble lithium salt is produced, which is then leached into the solution for additional purification. Based on the type of salt used, this process can be broadly categorized into four types: sulfate roasting [12,13,14], carbonate roasting [53,54], chloride roasting [55,56], and mixed salt roasting.

##### Sulfate Roasting Method

The primary sulfate utilized for lithium pyroxene is potassium sulfate. Conversely, for lithium mica, various sulfate salts are typically employed for mixed roasting with the ore. The corresponding reaction mechanism is depicted in Equations (1) and (2) [14,57,58,59,60]. In their study, Qunxuan Yan et al. [14] employed a sulfate roasting-water leaching lithium extraction process to extract lithium from lithium mica. Their findings indicated that both the roasting temperature and the mass ratio of Na_2_SO_4_ to additives (K_2_SO_4_ and CaO) exerted a significant influence on the extraction rate of lithium. The optimal process parameters were as follows: a reaction time of 0.5 h, a roasting temperature of 850 °C, and a mass ratio of lithium mica/Na_2_SO_4_/K_2_SO_4_/CaO of 1:0.5:0.1:0.1. These conditions yielded a lithium extraction rate of 91.61%. The resulting leaching solution contains trace amounts of impurity elements such as Al and Si, which facilitate the purification of lithium and align better with industrial production standards.

Li_2_O·Al_2_O_3_·SiO_2_ + K_2_SO_4_→K_2_O·Al_2_O_3_·3SiO_2_ + Li_2_SO_4_ (1)

Li_2_O·Al_2_O_3_·SiO_2_ + CaSO_4_→CaO·Al_2_O_3_·3SiO_2_ + Li_2_SO_4_ (2)

##### Carbonate Roasting Method

The carbonate roasting method, one of the earliest techniques for extracting lithium from ore, begins with the crushing and blending of limestone and lithium ore in a 3:1 mass ratio. The mixture is then subjected to high temperatures of 900 °C for roasting. Subsequently, the sintered block undergoes a series of processes including leaching, sedimentation, and separation to remove impurity ions such as Ca, Al, and Si. Finally, lithium precipitation is achieved by adding sodium carbonate, resulting in the formation of lithium carbonate precipitate. Equation (3) [61] illustrates one of the key reaction principles involved.

Li_2_O·A1_2_O_3_·4SiO_2_ + 8CaO→Li_2_O·Al_2_O_3_ + 4 [2CaO·SiO_2_] (3)

This method, while featuring a simple production process and economical raw materials, faces notable drawbacks. These encompass increased material circulation, higher energy consumption for evaporation, elevated slag production, a diminished lithium recovery rate, and various shortcomings. As a result, it is nearing obsolescence.

##### Chlorination Roasting Method

The chlorination roasting method aims to produce lithium chloride through the reaction of lithium ore with a chlorinating agent under specific conditions. Subsequently, the solution containing lithium ions is separated through water leaching. Chlorinating agents are categorized into gas and solid forms. Gas chlorinating agents encompass chlorine-containing substances like Cl_2_, HCl, and CCl_4_, while solid chlorinating agents primarily consist of chlorine-containing salts such as CaCl_2_. This method is classified into low-, medium-, and high-temperature roasting based on the temperature variation. Lithium mica ore serves primarily as a solid chlorinating agent, while various chlorine salts are utilized as roasting agents. High-temperature roasting of lithium pyroxene typically employs Cl_2_ or CaCl_2_ and mixed chloride salts. This approach offers advantages such as reduced roasting time and simplicity of process. Due to chlorine’s corrosive nature in the roasting process, equipment sustains more damage, leading to environmental pollution. This method has not yet been extensively adopted in industrial production. Further improvements in process conditions are necessary to determine the optimal dosage of the chlorinating agent.

##### Mixed Salt Roasting Method

The mixed salt roasting method involves blending various salts in specific proportions to serve as a roasting agent. For instance, the addition of ammonium sulfate during sulfate roasting can lower the roasting temperature and offer benefits such as reducing impurity ions.

##### Comparative Summary of Salt Processes

The sulfuric acid and carbonate roasting methods are cost-effective for lithium extraction. However, they generate a significant amount of waste material, like slag, increasing energy consumption. Additionally, the sulfuric acid method poses an environmental pollution risk due to harmful byproduct release.

In contrast, the chlorination roasting method produces less waste and achieves a high lithium conversion rate. Yet, it faces challenges like severe equipment corrosion and intricate chloride ion removal, affecting efficiency and sustainability.

Conversely, the mixed roasting method addresses some limitations of acid-based approaches but complicates the process flow. It also struggles with impurity removal, hindering achieving desired lithium purity levels.

#### 2.1.4. Pressure-Cooking Process

The pressure-cooking process involves treating ore and a salt solution in a high-pressure kettle to initiate a reaction. The main principle behind this process is the ion exchange, where alkali metal ions such as potassium and lithium dissolve to form salts. In the autoclaving process, which is commonly employed with lithium pyroxene and soda ash, high temperature and pressure conditions facilitate the ion exchange between lithium ions in the ore and sodium ions in the soda ash solution. This results in the precipitation of lithium carbonate, which is then further purified to obtain battery-grade lithium carbonate. The main reaction principle is illustrated in Equation (4) [62,63,64]. Due to its fluorine content, lithium mica requires roasting and defluorination with water vapor at temperatures ranging from 870 °C to 930 °C before autoclaving. Subsequently, a mixture of alkali, chlorine salt, sulfate, and carbonate, in specific proportions, is combined with the defluorinated material. This mixture is then placed into a high-pressure reactor to produce the lithium-containing leaching solution.

Li_2_O·Al_2_O_3_·4SiO_2_ + nH_2_O + Na_2_CO_3_→Na_2_O·Al_2_O_3_·4SiO_2_·nH_2_O + Li_2_CO_3_ (4)

#### 2.1.5. Summary of Lithium Resources from Ores

In summary, lithium extraction from ore presents various methods. While the acid process is straightforward, its corrosive nature demands specialized equipment and produces environmentally harmful acid mist. The alkali and pressure-cooking methods partially mitigate pollution concerns but require significant equipment investment and involve high alkali consumption, leading to low product purity. On the other hand, the salt process, though simple and efficient in lithium conversion, is prone to introducing impurity ions, impacting product quality. The lithium mine faces shrinking ore resources, heightening the importance of exploring lithium extraction from salt lakes.

### 2.2. Salt Lake Brine Lithium Extraction Process

Various methods exist for extracting lithium from salt lake brine, including chemical precipitation, solvent extraction, electrochemical processes, membrane techniques, and adsorption.

#### 2.2.1. Chemical Precipitation Method

**Figure 5:**
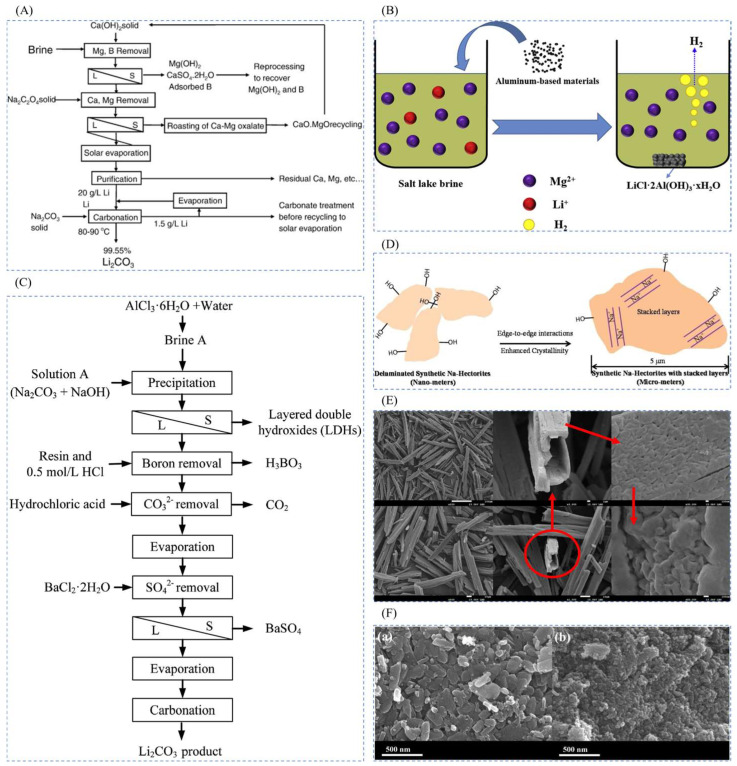
(**A**) Flowchart of the process developed to recover lithium as a carbonate from Uyuni brine [65], reproduced with permission from *Elsevier*, 2012; (**B**) schematic diagram of the aluminate precipitation method [35], reproduced with permission from *Elsevier*, 2018; (**C**) flowchart of the co-preparation of layered double hydroxides (LDHs) and lithium carbonate [67], reproduced with permission from *Elsevier*, 2018; (**D**) co-precipitation [68], reproduced with permission from *Elsevier*, 2018; (**E**) co-precipitation with hydrothermal duration [69], reproduced with permission from *Elsevier*, 2022; (**F**) SEM images of the initial Li^+^ concentration: (**a**) 1000 mg·L^−1^ and (**b**) SEM images of Li_3_PO_4_ at 3000 mg·L^−1^ [70], reproduced with permission from *Elsevier*, 2022.

The chemical precipitation method relies on harnessing solar energy for the natural evaporation and concentration of salt lake brine. By introducing a precipitant, the brine undergoes a process where various interfering ions, including Mg^2+^, Ca^2+^, and B^3+^, precipitate. Eventually, the targeted Li^+^ ions settle, resulting in the extraction of purer lithium resources. Chemical precipitation methods are mainly divided into carbonate precipitation as is shown in Figure 5A [65], aluminate precipitation as is shown in Figure 5B [66], phosphate precipitation [16], magnesium precipitation as is shown in Figure 5C [67], co-precipitation as is shown in Figure 5D,E [68], etc. Typically, Li^+^ ions are precipitated as LiHCO_3_, Li_2_CO_3_, or Li_3_PO_4_. The primary precipitating agents include sodium bicarbonate, sodium carbonate, sodium phosphate, or sodium fluoride.

Zhang et al. [69] used Struvite Precipitation to separate lithium and magnesium by triammonium phosphate trihydrate precipitation, and investigated the effects of parameters such as initial pH, stirring rate, dosage of triammonium phosphate trihydrate, aging time, and initial Li^+^ and Mg^2+^ concentrations; solution chemistry calculations, DFT calculations, XRD, SEM, BET, and zeta potential measurements were used to study the related separation and adsorption mechanism. The best conditions to precipitate Li^+^ were obtained when pH = 5.70, stirring rate 300 r·min^−1^, dosage of triammonium phosphate trihydrate 760 g·L^−1^, aging time 0 min, c(Li^+^) = 2 g·L^−1^, c(Mg^2+^) = 60 g·L^−1^, and the conclusion was obtained through chemical calculations that the triammonium phosphate can be applied to two kinds of real salt lake brines, which show the excellent separation performance.

Junho Shin et al. [70] used waste lithium solution as a raw material to prepare concentrated lithium solution by an integrated precipitation-reflow process, which separates lithium ions into Li_3_PO_4_ by precipitation, and then converts it into lithium solution by reflux reaction with metal chloride solution. The method here is simple and environmentally friendly and does not require acid leaching of insoluble lithium compounds to prepare concentrated lithium solution. The method improves the process efficiency of producing high-purity lithium carbonate from waste lithium solution as is shown in Figure 5F and is economically feasible. Zhang et al. [71] proposed a new coupled biofilm-precipitation system to investigate the feasibility of separating Mg^2+^ from brine for the recovery of Li^+^.

#### 2.2.2. Solvent Extraction Method

Solvent extraction is a primary method for selectively separating and extracting metal ions. It exploits differences in solubility or partition coefficients between solutes in aqueous and organic phases. This facilitates the transfer of solutes from the aqueous phase to the organic phase, where solubility for the solute is greater, achieving solute trans-phase separation [72,73]. Commonly studied extractants include neutral phosphorus-containing compounds [20,74] and β-bis(ketone) extractants [75]. Examples of neutral phosphorus-containing extractants are tributyl phosphate (TBP), dibutylphosphonate (BDBP), and tri-n-octylphosphine oxide (TOPO).

**Figure 6:**
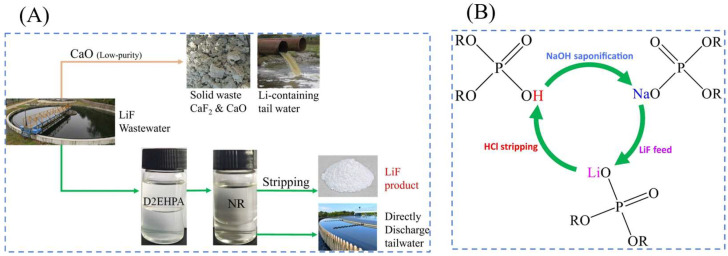
(**A**) Schematic diagram of recovery [36]; (**B**) mechanism of D2EHPA extract of Li^+^ [36]. Reproduced with permission from *Elsevier*, 2023.

Han et al. [36] proposed an ideal route as is shown in Figure 6 for the recovery of Li^+^ and F^−^ by di(2-ethylhexyl) phosphate (D2EHPA) and neutral solvents. A mixed neutral solvent (NR) extraction and stripping (with HCl) based on TOPO/CYANEX923 (mixed tri-n-alkylphosphine oxide)/TBP was systematically investigated to adjust the ionic morphology, followed by extraction and stripping of F^−^. A concentrated solution of LiCl and LiF crystals were designed to be produced by stripping the aqueous solution with high Li^+^ and F^−^ recoveries.

Zhang et al. [76] proposed a process for the recovery of lithium from South American salt lakes by liquid–liquid extraction using β-bis(ketone) system as the extractant. The extraction system was optimized by comparing the effects of TBP, trialkylphosphine oxide (TRPO), D2EHPA, and phosphoric acid-2-ethylhexyl-2-ethylhexyl ester (P507) on the extraction of the metal, and the extraction system was finally determined as 3-benzoyl-1,1,1-trifluoroacetone/trialkylphosphine oxide (HBTA/TRPO). The selectivity mechanism of the HBTA/TRPO system for metal ions was illustrated using DFT calculations, and the optimized structures of the complexes formed by Li^+^, Na^+^, K^+^, Ca^2+^, Mg^2+^ and HBTA/TRPO were obtained. A new method is provided for the efficient recovery of high sodium–lithium ratio salt lake brine.

#### 2.2.3. Electrochemical Method

Electrochemical lithium extraction, an emerging technology inspired by the operational principles of lithium iron phosphate batteries, offers notable advantages such as strong selectivity, high adsorption efficiency, and environmental friendliness. This innovative approach is a focal point in academic research. The process involves embedding, detaching, and transferring Li^+^ ions within the electrode material, electrolyte, and brine of the salt lake, controlled by the potential. The cyclic enrichment process facilitates the extraction of lithium from the salt lake brine, marking a significant step in sustainable lithium recovery. 

**Figure 7:**
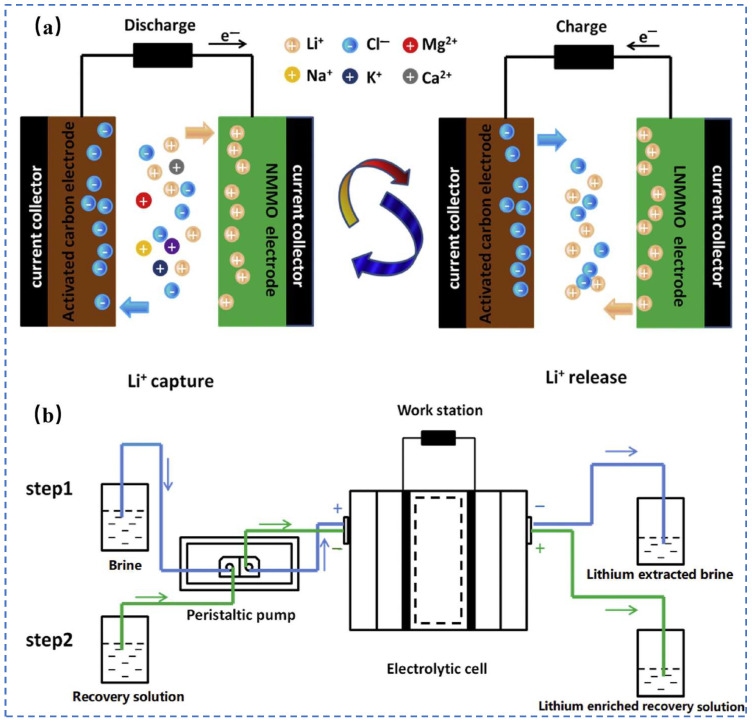
(**a**) Schematic diagram of the NMMO/AC hybrid ultra-capacity electrochemical process for lithium recovery and release. (**b**) Diagram of the semi-continuous recovery process [21]. Reproduced with permission from *Elsevier*, 2020.

Zhao et al. [21] synthesized LiNi_0.03_Mo_0.01_Mn_1.96_O_4_(LNMMO) as the anode material, assembled a hybrid supercapacitor consisting of an LNMMO cathode and an AC anode, and combined the LNMMO/AC hybrid supercapacitor with a self-designed continuous flow control system to develop a continuous flow LNMMO/AC hybrid supercapacitor (CF-LNMMO/AC) as is shown in Figure 7 for selective trapping of Li^+^ in a 30 mM mixed cation solution and a simulated solution of Salar de Uyuni salt lake brine.

#### 2.2.4. Membrane Method

The membrane separation method represents an innovative approach to separation technology, utilizing membranes as the separation medium to effectively isolate and purify impurity ions within a solution [33,77]. The fundamental principle revolves around leveraging the microporous and microchannel structures inherent in polymer materials. These structures enable the separation of small molecules or ions from the solution through the membrane, achieving the distinct separation and purification of various substance components within the solution. This method encompasses different techniques, including electrodialysis, nanofiltration, membrane extraction, ion-imprinted membrane, and more. Each variant plays a crucial role in achieving specific separation and purification objectives within diverse contexts.

**Figure 8:**
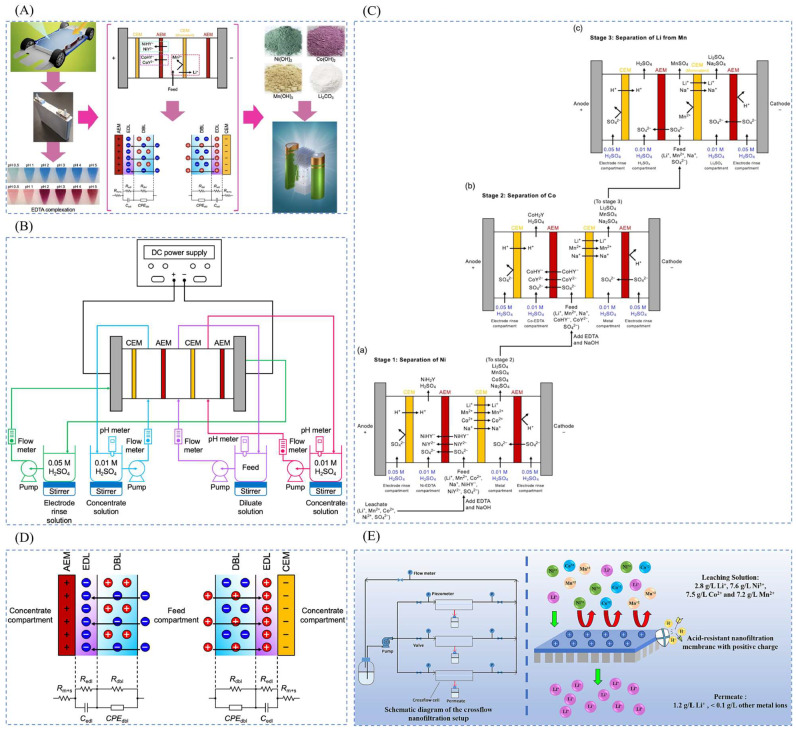
(**A**) Diagrammatic representation of the application of ED [37], reproduced with permission from *Elsevier*, 2022; (**B**) schematic diagram of the experimental setup for electrodialysis for the separation of Li, Ni, Mn, and Co from NMC111 chemistry [37], reproduced with permission from *Elsevier*, 2022; (**C**) principle of the three-stage electrodialysis process for the separation of Li, Ni, Mn, and Co from NMC111 chemistry. (**a**) Separation of Ni in stage 1. (**b**) Second stage Co separation. (**c**) Separation of Li from Mn in stage 3 [37], reproduced with permission from *Elsevier*, 2022; (**D**) diagram of resistive layer and equivalent circuit modeling [37], reproduced with permission from *Elsevier*, 2022; (**E**) diagram of nanofiltration membrane operation [81], reproduced with permission from *Elsevier*, 2024.

Electrodialysis (ED) is a membrane separation process using ion exchange membrane as the medium and potential difference as the driving force [78]. As a relatively mature technology in membrane separation process, electrodialysis has been widely used in water treatment process. The study of lithium concentration of high magnesium–lithium ratio brine using ion exchange membranes with selectivity for monovalent ions and the mother liquor after lithium extraction can be recycled. ED is further classified into selective electrodialysis [75], bipolar membrane electrodialysis [79], and click electrodialysis [80]. Chen et al. [37] developed a novel ED process using electrodialysis to separate Li, Ni, Mn, and Co from LiNi_0.33_Mn_0.33_Co_0.33_O_2_ (NMC111), as is shown in Figure 8A–D.

The principle of nanofiltration (NF) separation is that NF membrane can retain bivalent and above metal cations, while monovalent lithium and sodium ions can pass through, so that lithium ions and magnesium ions can be separated from the old brine in the potassium extraction method. A two-stage nanofiltration method was used to separate Mg^2 +^ and Li^+^ from the brine of a salt lake, and a reverse osmosis membrane was used to enrich the lithium-containing solution. After the nanofiltration membrane separation operation, the permeate water can be used as feed water for the reverse osmosis membrane to produce lithium salts, and the concentrated water can be used to extract magnesium salts, so that the inorganic salt resources in the brine of the salt lake can be comprehensively utilized. Gao et al. [81] tested and characterized two commercial acid-resistant NF membranes (NF270 and DK) to understand their structures and differences as is shown in Figure 8E. By evaluating the performance of these NF membranes in terms of separation efficiency, selectivity, and permeate flux, an innovative and sustainable method for the recovery of Li^+^ was provided.

#### 2.2.5. Adsorption Method

The adsorption method employs a selective adsorbent for lithium ions, binding them before eluting and extracting under the influence of an eluent. This process effectively separates lithium ions from other impurity ions. Based on the adsorbent’s nature, categories include organic ion-exchange adsorbents, inorganic ion-exchange adsorbents, and novel materials. These categories rely on inherent characteristics for lithium ion adsorption, facilitating improved separation from other impurities. Examples of organic adsorbents encompass chelating resin and sulfonic acid resin. Inorganic adsorbents consist of aluminosilicate, heteropoly acid, metallate, and ferrocyanide. Additionally, there are new materials like composite adsorbents.

#### 2.2.6. Summary of Lithium Resources from Salt Lakes

The salt lake brine lithium extraction process is cost-effective, saving energy and expenses compared to traditional ore extraction methods. Moreover, it is environmentally friendly, minimizing damage to the geological environment and avoiding the substantial waste associated with ore mining. This extraction process boasts high efficiency and productivity, enabling large-scale lithium production. In the lithium extraction process from salt lake brine, the presence of other minerals like magnesium and potassium necessitates additional separation and treatment steps, thereby increasing complexity and costs. Furthermore, this extraction method is constrained by geographic and climatic conditions, restricting its applicability to specific regions. Additionally, due to the comparatively low lithium content in salt lake brine, extensive treatments are required to achieve commercially viable extraction levels, potentially making it less economical for small-scale production or brines with low lithium concentrations.

### 2.3. Summary

The conventional lithium extraction processes have become increasingly inadequate for meeting the demands of lithium extraction across various environments. Consequently, there is a growing need for novel extraction methods tailored to different lithium extraction settings in China. While numerous studies explore various techniques for extracting lithium from diverse sources, these approaches often exhibit low selectivity for Li+ adsorption. In contrast, lithium ion-imprinted materials offer distinct advantages for the precise extraction of lithium ions. Utilizing ion-imprinting technology, these materials can achieve targeted adsorption of specific ions. Hence, preparing lithium ion-imprinted polymers through ion-imprinting technology is an optimal strategy for adsorbing lithium ions across various environments.

## 3. Synthesis Method of Lithium Ion-Imprinted Materials and Related Introduction

**Figure 9:**
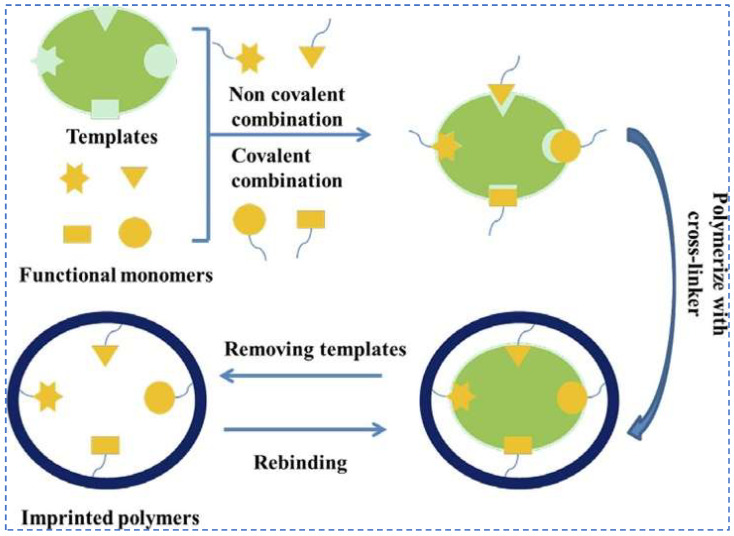
Schematic diagram of the synthesis process of IIP [82]. Reproduced with permission from *Elsevier*, 2022.

Lithium ion-imprinting technology is a subset of ion-imprinting technology. It involves the preparation of polymers characterized by high selectivity and affinity for target ions, boasting three key attributes: target extraction, specific recognition, and broad applicability. The resultant polymers from this process are referred to as lithium ion-imprinted polymers (Li-IIPs). Figure 9 illustrates the fundamental synthesis steps of Li-IIPs. Firstly, Li^+^ is complexed with a complexing agent to form a new template ion, and then monomer molecules with a certain recognition function and template ions are chemically combined together for prepolymerization to form a prepolymer. Then, a cross-linking agent is added, and then the polymerization reaction is initiated under the action of the initiator, so as to form a porous polymer with a cross-linked structure, and finally the lithium ion-imprinted material is prepared by eluting and removing Li^+^. The lithium ion-imprinted polymer is insensitive to the external environment, can be stabilized in high temperature, strong acid, strong alkali, organic phases and other harsh conditions, and the preparation is simple, low cost, and can be reused.

This chapter primarily covers five key aspects: (1) introducing the system for preparing lithium ion-imprinted materials; (2) describing the utilization of chemical calculations to select the suitable complexing agent; (3) elaborating on conventional methods employed in the preparation of lithium ion-imprinted materials; (4) outlining methods for characterizing these materials; and (5) introducing fitting equations for evaluating the properties of the materials.

### 3.1. System of Lithium Ion-Imprinted Materials

**Figure 10:**
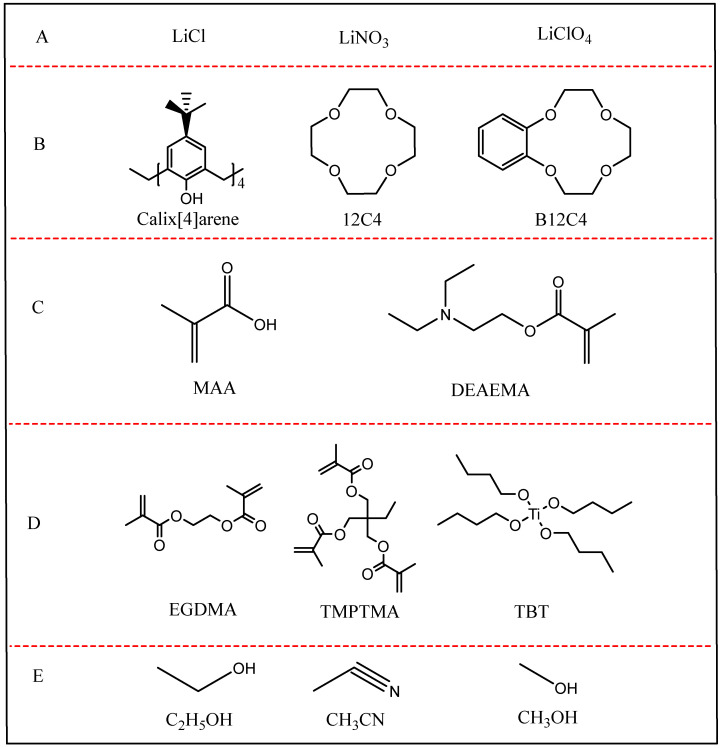
(**A**) Template ions; (**B**) complexing agent; (**C**) functional monomer; (**D**) cross-linking agent; (**E**) solvent [38,39,40,41,83,84,85,86,87].

The system of lithium ion-imprinted material mainly includes template ion, complexing agent, functional monomer, cross-linking agent, initiator, solvent, and so on. A, as is shown in Figure 10. Template ions choose Li^+^, mainly LiCl, LiNO_3_, LiClO_4_, etc. [38]; complexing agents include cup [4] aryl hydrocarbons, crown ethers, benzo-12-crown-4 (B12C4), etc. [39]; functional monomers mainly include methyl methacrylate (MAA), diethylaminoethyl methacrylate (DEAEMA), etc. [83,84]; cross-linking agents commonly used are ethylene glycol dimethacrylate (EGDMA) [40,41], trimethylolpropane trimethacrylate (TMPTMA) [85], tetrabutyl titanate (TBT), triethyleneglycol dimethacrylate, and pentaerythritol triacrylate, etc.; the most common initiator is azobisisobutyronitrile (AIBN) [86]; solvents are generally of low polarity, and they are mostly in anhydrous ethanol (C_2_H_5_OH), acetonitrile (CH_3_CN), methanol (CH_3_OH) and dimethylformamide (DMA) [87].

### 3.2. Quantum Chemical Computing for Lithium Ion-Imprinted Materials

**Figure 11:**
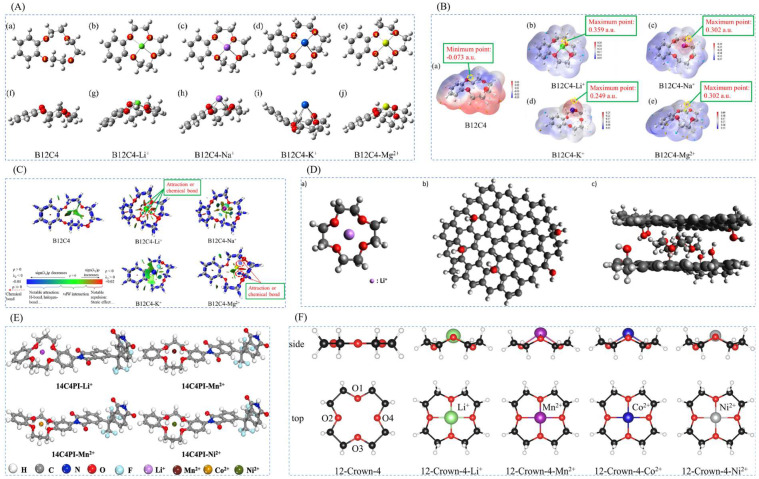
(**A**) Top view of the optimized structures of (**a**) B12C4 and its complexes with (**b**) Li^+^, (**c**) Na^+^, (**d**) K^+^ and (**e**) Mg^2+^; (**f**) B12C4, (**g**) B12C4- Li^+^, (**h**) B12C4- Na^+^, (**i**) B12C4- K^+^ and (**j**) B12C4- Mg^2+^ in side view [31]; (**B**) ESP polar distributions of (**a**) B12C4, (**b**) B12C4- Li^+^, (**c**) B12C4- Na^+^, (**d**) B12C4- K^+^ and (**e**) B12C4- Mg^2+^ [31]; (**C**) interaction visualization plots of B12C4 and its complexes [31]; (**D**) optimized molecular geometries of the (**a**) lithium ion/12C4 ensemble, (**b**) graphene nanosheet (partially oxidized), and (**c**) 12C4/graphene nanosheets ensemble [88]; (**E**) optimized geometries of 14C4PIs with chelated metal ions; (**F**) geometry of the crown-ligand-metal complexes shapes shown from side and top views [89]. Reproduced with permission from Ref. [31], *Elsevier*, 2023; [88], *Elsevier*, 2023; [89], *Elsevier*, 2021.

Quantum chemical computing of density functional theory is used to provide a scientific and theoretical support point for the selection of suitable complexing agents [31,88,89]. As is shown in Figure 11, the main calculation is to calculate the coordination of the complexing agent and template ions to verify the stability of the structure; the ESP of the complexes was calculated by Multifwn 3.8 program or other programs, and then electrostatic potential analysis was carried out; the interaction force between the complexing agent and the template ions can also be calculated, and the calculations, such as analyzing the principle of spatial potential resistance effect complexing agent and template ions, are verified through a series of quantum chemical computing the high selectivity of the lithium ion-imprinted material and the targeted extraction of lithium.

### 3.3. Introduction to Conventional Methods

The synthesis mechanisms of lithium ion-imprinted materials involve mechanisms such as the sol-gel method and free-radical polymerization. Conventional synthesis methods encompass suspension polymerization, precipitation polymerization, and native polymerization. Additionally, emerging technologies, including surface imprinting technology, column separation technology, magnetic separation technology, membrane separation technology, and electrochemistry, are integrated with conventional methods. These diverse approaches are employed to prepare various types of materials.

#### 3.3.1. Synthesis Mechanism

**Figure 12:**
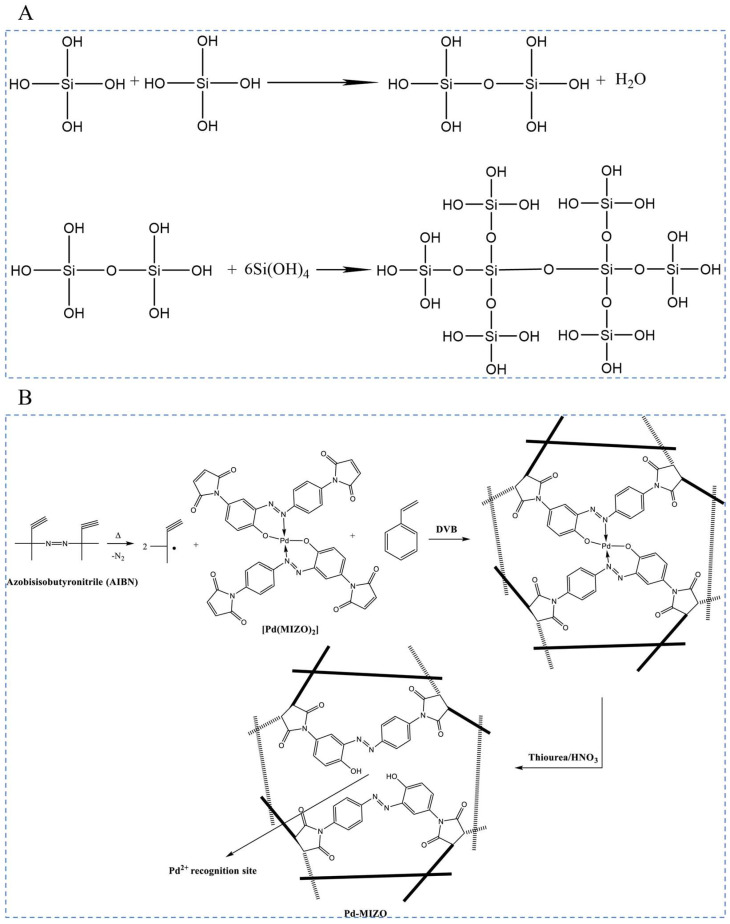
(**A**) Sol-gel process to form the 3D network structure of Cu(II) ion-imprinted polymers [90]; (**B**) synthesis process of ion-imprinted materials of palladium (free-radical polymerization) [29].

The sol-gel method uses compounds containing highly chemically active components as precursors; these raw materials are uniformly mixed in the liquid phase, and hydrolysis, condensation chemical reaction, the formation of a stable transparent sol system in solution, the sol is slowly polymerized by the aging of the gel particles between the gel to form a three-dimensional network structure of the gel, and the gel network is filled with solvent that has lost its mobility to form a gel [90] as is shown in Figure 12A. The gels are dried and sintered and cured to prepare molecular and even nanosubstructured materials. Free-radical polymerization is a polymer chemical reaction initiated by free radicals, starting with an initiation phase where the initiator generates free radicals and these radicals initiate the joining of monomers. The transfer phase results in the gradual extension of the chains to form polymer chains [29] as is shown in Figure 12B. This process is flexible and applicable to a wide range of monomers to prepare polymers such as polyethylene and polystyrene. However, its molecular weight distribution is wide and difficult to control. The termination phase can be realized by free radical encounter or otherwise. Free-radical polymerization is widely used in industry and provides a viable route to synthesize a variety of plastics and rubbers.

#### 3.3.2. Conventional Methods

**Figure 13:**
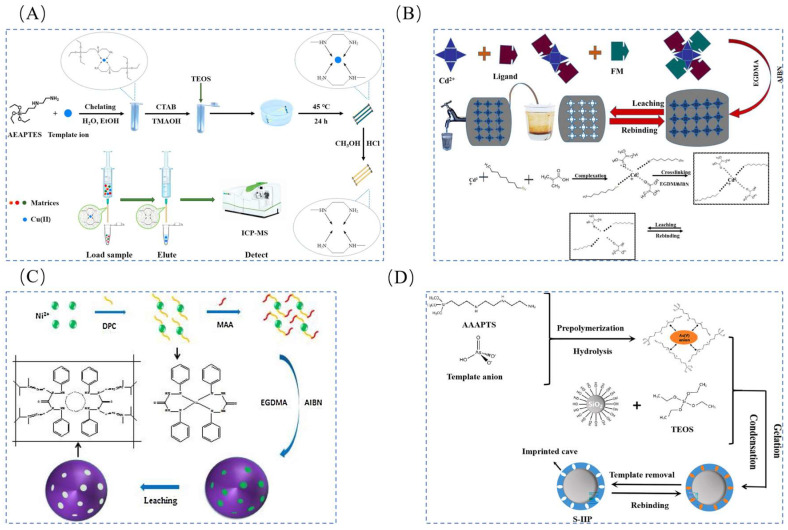
(**A**) Preparation of Cu(II) ion blotting hybrid monolithic column and SPME-ICP-MS analysis procedure by suspension polymerization [28,91]; (**B**) preparation of Cd(II) ion-imprinted polymers by precipitation polymerization [91,92]; (**C**) preparation of Ni(II)-imprinted polymers by bulk polymerization [93,94]; (**D**) process flow chart for the preparation of As-IIP by surface-imprinted polymerization [95]. Reproduced with permission from Ref. [28], *Elsevier*, 2021; [91], *Elsevier*, 2010; [93], *Nature*, 2014; [94], *Elsevier*, 2018; [95], *Elsevier*, 2022.

Suspension polymerization is a method wherein functional monomers, cross-linking agents, and initiators are initially dissolved in an organic solvent. This organic phase is then added to water, resulting in the formation of dispersed and stable droplets under high-speed stirring. Subsequently, the polymerization reaction is triggered at a specific reaction temperature, leading to the production of the imprinted polymer [28,91] as shown in Figure 13A. Precipitation polymerization entails dissolving template ions and functional monomers in organic solvents in a specific ratio. Following this, a measured quantity of cross-linking agent and initiator is added to initiate the polymerization reaction, resulting in the formation of polymer precipitates [91,92] as shown in Figure 13B. Intrinsic polymerization includes adding a soluble initiator to the pure monomer in liquid form, with the initiator chosen to be soluble in the system comprising the monomer, cross-linker, and solvent. The exothermic polymerization process typically spans several hours for completion. Following this, the resulting polymer undergoes additional operations like milling. Despite these processes, the synthesized particles retain a substantial molecular weight and size [93,94] as shown in Figure 13C. Many lithium ion-imprinted materials today are prepared using a combination of novel techniques and traditional methods, rather than relying solely on a single polymerization approach. Surface modification technology, particularly surface imprint polymerization, is widely utilized. This method aims to diminish the buildup of adsorption sites by creating sites with a larger specific surface area on the carrier’s surface. By enhancing the number of effective adsorption sites, it addresses challenges such as deep embedding, limited mass transfer, and difficulties in regeneration [95] as shown in Figure 13D. Additionally, column separation, magnetic separation, membrane separation, electrochemistry, and other techniques are also employed in combination.

### 3.4. Characterization of Lithium Ion-Imprinted Materials

**Figure 14:**
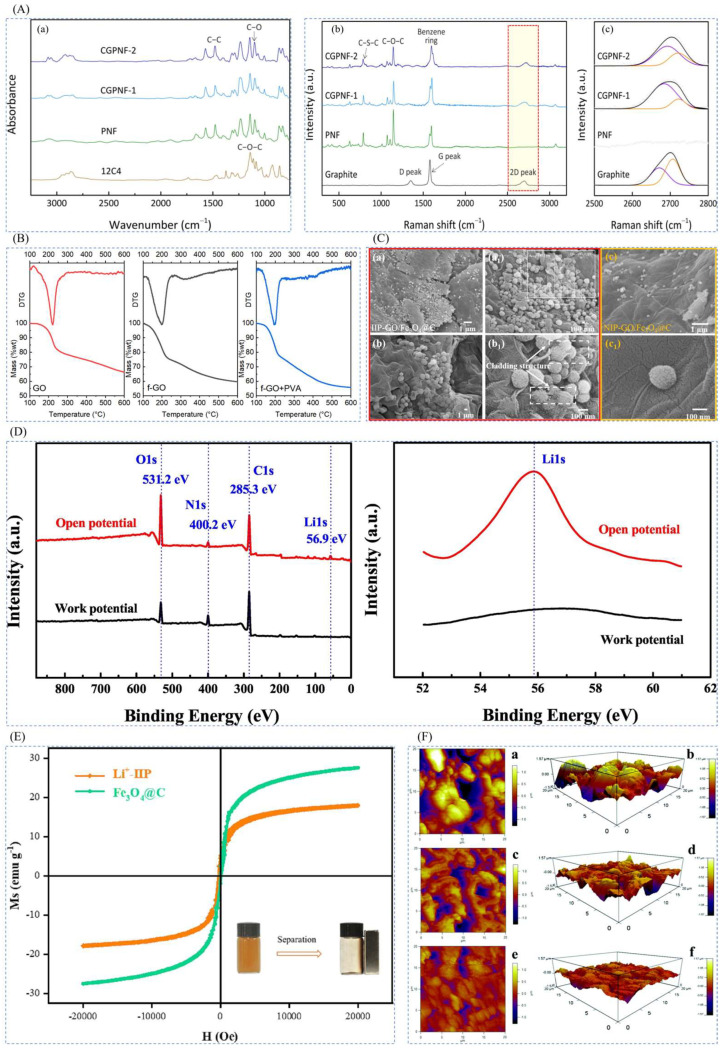
(**A**) (**a**) FT-IR spectra of CGPNF-2 membrane, CGPNF-1 membrane, PNF membrane and 12C4, (**b**,**c**) Raman spectra of CGPNF (CGPNF-1 and CGPNF-2) membranes, PNF membranes, and graphite flakes: full spectra, zoomed-in spectra of the 2D peak region [88]; (**B**) FT-IR spectra of f-GO membrane, nanocomposite membranes, and nanocomposite (f-GO+PVA) membranes, three membranes with TGA and DTG; (**C**) FESEM images of IIP-GO/Fe_3_O_4_@C ((**a_1_**) and (**b_1_**) are magnified SEM images of (**a**) and (**b**), respectively) and NIP-GO/Fe_3_O_4_@C ((**c_1_**) is a magnified SEM image of (**c**)) [32]; (**D**) XPS spectra of Li-IIM at the figure of merit and open potentials [42]; (**E**) Magnetization curves of Fe3O4@C and Li-IIM, and the magnetic separation process of Li-IIM are shown in the lower right corner [31]; (**F**) AFM images of GO/PVDF (**a**,**b**) pDA@GO/PVDF (**c**,**d**) and LIHM (**e**,**f**) [41]. Reproduced with permissions from Ref. [31], *Elsevier*, 2023; [32], *Elsevier*, 2021; [41]; *RCS Publishing*, 2017; [88], *Elsevier*, 2023; [42], *Elsevier*, 2020.

There are many conventional characterization methods, as is shown in Figure 14, including spectroscopy methods (infrared spectroscopy IR and ultraviolet UV-Vis), nuclear magnetic resonance (proton nuclear magnetic resonance ^1^H-NMR and carbon-13 nuclear magnetic resonance ^13^C-NMR), mass spectrometry methods (proton mass spectrometry), thermal analysis methods (differential scanning gauge DSC and thermogravimetric analysis TGA), surface analysis methods (scanning electron microscopy (SEM and X-ray photoelectron spectroscopy XPS), crystallography methods (X-ray diffraction XRD), electrical and magnetic methods (conductivity measurement and magnetic measurement), chromatography (gas chromatograph GC and liquid chromatography HPLC), etc. The most common of these are IR, SEM, XRD, etc.

### 3.5. Performance Evaluation of Lithium Ion-Imprinted Materials

To assess material properties, scientific computational fitting is essential for validating experimental results. This involves applying equations, commonly found in Table 1, to perform the fitting process [31,32].

## 4. Different Types of Lithium Ion-Imprinted Materials

This chapter highlights the research advancements in various lithium ion-imprinted polymers. These polymers are developed through the integration of novel technologies and conventional methods to extract lithium from salt lake brines. The materials can be classified into four categories based on the technology employed: lithium ion-imprinted adsorbent columns, lithium ion-imprinted magnetic adsorbent materials, lithium ion-imprinted adsorbent membrane materials, and other types of materials.

### 4.1. Lithium Ion-Imprinted Adsorption Columns

The lithium ion-imprinted adsorption column involves molding the imprinted material into a packed column or placing it on top of the column. This configuration facilitates convenient adsorption and separation operations. Molding the imprinted material into a column eliminates the need for tedious separation and recovery procedures post-adsorption.

#### 4.1.1. Preparation of Imprinted Adsorption Columns by Precipitation Polymerization Method

**Figure 15:**
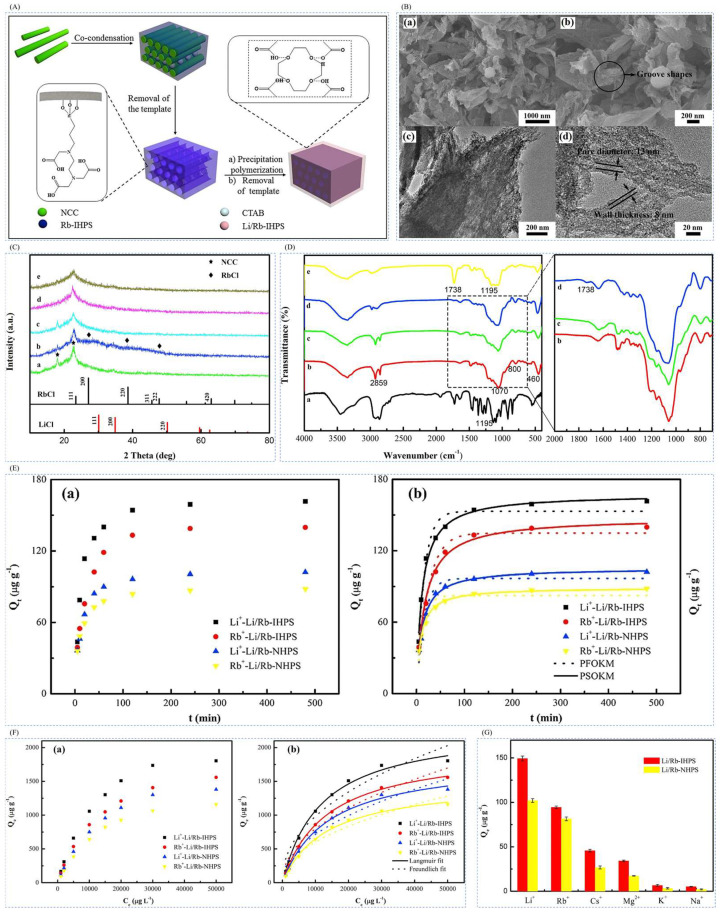
(**A**) Synthesis route of Li/Rb-imprinted multistage mesoporous silica (Li/Rb-IHPS); (**B**) SEM images of Li/Rb-IHPS (**a**,**b**) and TEM images of Li/Rb-IHPS (**c**,**d**); (**C**) RbCl, LiCl, NCC/multistage mesoporous silica (HPS) composites (a), NCC/Rb-IHPS composites (b), Rb-IHPS (c), XRD patterns of unimpregnated Li/Rb-IHPS (d), and Li/Rb-IHPS (e); (**D**) XRD patterns of 12C4 (a) NCC/multistage mesoporous silica (HPS) composites (b), NCC/Rb-IHPS composites (c), Rb-IHPS (d), and Li/Rb-IHPS (e) Fourier transform infrared spectra; (**E**) (**a**) effect of contact time on Li+ and Rb+ adsorption of Li/Rb-IHPS and Li/Rb-NHPS, and (**b**) unlined fitting curves for the proposed primary and quasi-secondary kinetic models; (**F**) (**a**) effect of Li^+^ and Rb^+^ initial concentration on the equilibrium adsorption capacity of Li/Rb-IHPS and Li/Rb-NHPS, (**b**) nonlinear fitting curves from Langmuir and Freundlich isotherm models; (**G**) adsorption capacity of Li/Rb-IHPS and Li/Rb-NHPS for Li^+^, Rb^+^, and other ions [30]. Reproduced with permission from *Elsevier,* 2018.

Xu et al. [30] prepared a novel Li/Rb imprinted multistage mesoporous silica (Li/Rb-IHPS) by precipitation polymerization in 2018 for the simultaneous extraction of Li^+^ and Rb^+^, in low concentrations. In this study, multistage mesoporous silica (HPS) was synthesized as a substrate material using natural biomaterial nanocrystalline cellulose (NCC) as a hard template and cetyltrimethylammonium bromide (CTAB) as a soft template by the dual template method. Rb^+^ imprinted multistage mesoporous silica (Rb-IHPS) was synthesized by the co-condensation method using tetraethoxysilane (TEOS) and N- [(3-trimethoxysilyl)propyl]silane. N- [(3-trimethoxysilyl) propyl] ethylenediaminetriacetic acid trisodium salt (TMS-EDTA) Li^+^-imprinted multistage porous mesoporous silica (Li-IHPS) was prepared by precipitation polymerization method using LiCl-H_2_O as template ion, 12-crown ether-4 (12C4) as complexing agent, MAA as functional monomer, EGDMA as cross-linking agent, and AIBN as initiator. The SEM and TEM in Figure 15B can clearly see the mesoporous microstructure of the sample; the XRD in Figure 15C can observe the characteristic peaks of the material due to judging the synthesis process of the material; Figure 15D,E show the adsorption kinetics and adsorption isotherm of the material, and the maximum adsorption capacity of the material was 2321.6 μg·g^−1^ for Li^+^, and the maximum adsorption capacity was 2321.6 μg·g^−1^ for Li^+^, and the high selectivity for Li^+^ can be seen in Figure 15F.

#### 4.1.2. Preparation of Imprinted Adsorption Columns by Surface-Imprinted Polymerization Method

**Figure 16:**
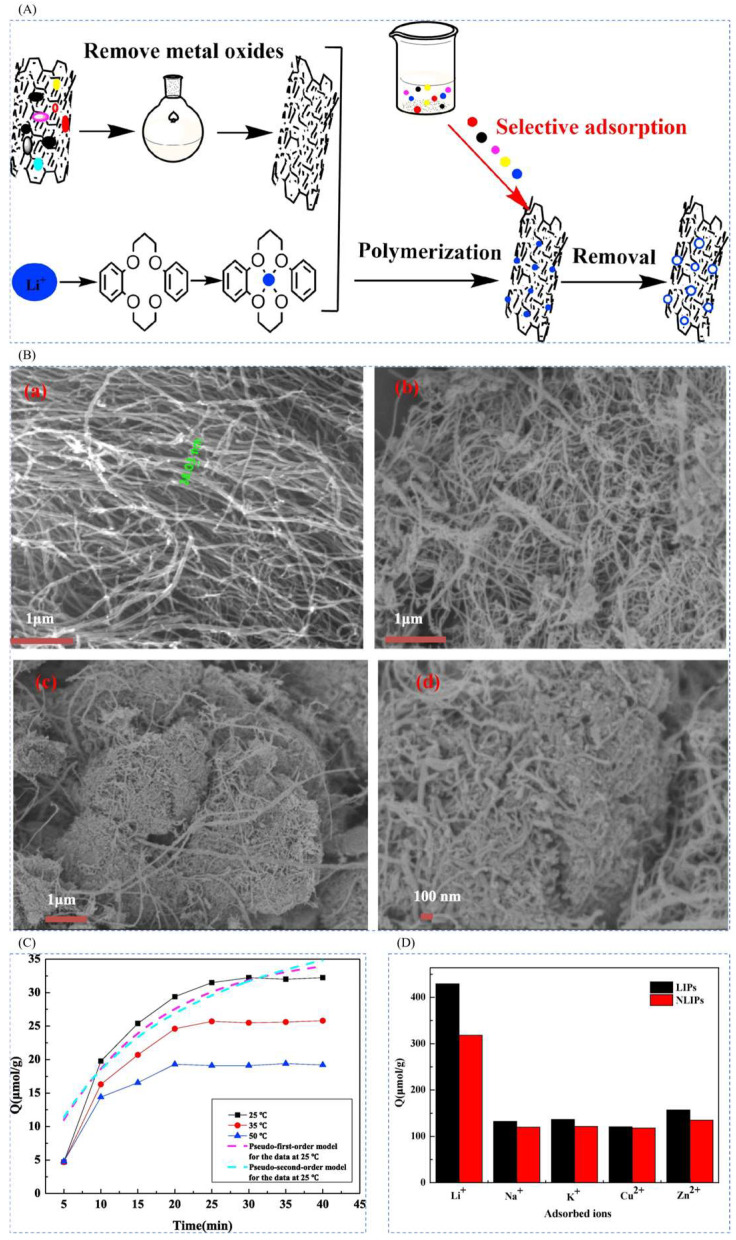
(**A**) Li^+^-IIP prepared by surface blotting; (**B**) SEM images of MWCNTs (**a**), NLi^+^-IIPs (**b**), and Li^+^-IIPs (**c**,**d**); (**C**) adsorption kinetics of Li^+^-IIP; (**D**) effect of interfering ions on the adsorption capacity of Li^+^ [84]. Reproduced with permission from *Elsevier*, 2018.

In order to extract lithium resources in water, Huang et al. [84] successfully prepared novel lithium ion-imprinted polymers (Li^+^-IIP) decorated on the surface of multi-walled carbon nanotubes by surface-imprinted technique, using LiNO_3_ as a template, dibenzo-14-crown-4 (DB14C4) as a complexing agent, MAA as a functional monomer, EGDMA as a cross-linking agent, and AIBN as an initiator. The lithium ion-imprinted materials Li^+^-IIPs were prepared, and the preparation process is shown in Figure 16A. SEM reveals that the multi-walled carbon nanotubes (MWCNTs) have a smooth surface and regular shape, and their tube diameters are about 38.03 nm; the maximal adsorption capacity of Li^+^-IIPs is 32.23 μmol·g^−1^; the Li^+^-IIPs and N Li^+^-IIPs have a better Li^+^ selectivity which was better than that of Na^+^, K^+^, Cu^2+^ and Zn^2+^, which proved that the selective separation of Li^+^ from aqueous solution could be realized by using DB14C4 as a chelating agent, reflecting the high selectivity of this material; the adsorption capacity of Li^+^-IIPs for Li^+^ did not decrease significantly with the increase of the cycling time. Compared with the first adsorption capacity, the adsorption capacity only decreased by about 10.3% after 10 cycles, indicating that Li^+^ -IIPs can be regenerated efficiently and its binding sites can maintain good adsorption stability. The results indicate that Li^+^-IIPs have good regeneration performance and have the potential to extract and recover Li^+^ from water.

#### 4.1.3. Electrochemical Preparation of Imprinted Adsorption Columns

**Figure 17:**
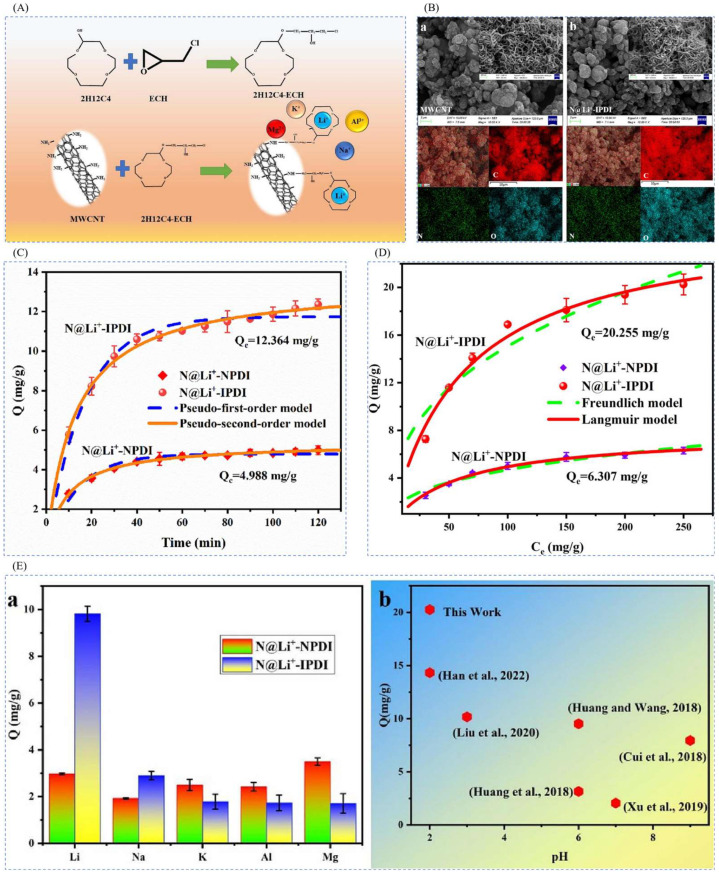
(**A**) Schematic of the synthesis of N@Li^+^-IPDI; (**B**) SEM (**a**) and EDS (**b**) scan images of MWCNT and N@Li^+^-IPDI electrode materials; (**C**) kinetic curve fitting of N@Li^+^-NPDI and N@Li^+^-IPDI; (**D**) isotherms of Li^+^ on N@Li^+^-NPDI and N@Li^+^-IPDI; (**E**) (**a**) ability of the N@Li^+^-NPDI and N@Li^+^-IPDI electrodes to selectively recover ions from competing ions; and (**b**) comparison of the present study with other works. Reproduced with permission from Ref. [43], *Elsevier*, 2023.

Ning et al. [43], in order to recover pure lithium resources in complex acidic solutions, immobilized 2H12C4 on the surface of multi-walled carbon nanotubes (MWCNTs) via the halogenation reaction of epichlorohydrin (ECH) using 2-hydroxymethyl-12-crown ether-4 (2H12C4) as a functional group to obtain the electrode material N@ Li^+^-IPDI for lithium ion selective recovery. Figure 17A shows the synthesis schematic, LiNO3 as template ion, 2H12C4 as complexing agent, ECH as functional monomer, acetic acid as cross-linking agent, and MWCNT as carrier to obtain 2H12C4-MWCNT, and then add acetylene black, polyvinylidene difluoride (PVDF), and N-methylpyrrolidone (NMP) to prepare the N@ Li^+^-IPDI. The SEM and EDS scans of the material are shown in Figure 17B, which shows that the three-dimensional lattice structure of MWCNT in this electrode material is more complete; the maximum adsorption capacity of the material is 20.255 mg·g^−1^ as can be seen from the adsorption kinetic fitting curves in Figure 17C and the isothermal adsorption line in Figure 17D, and the adsorption capacity of N@ Li^+^-IPDI is higher than that of the other ions as can be seen from Figure 17E.

### 4.2. Lithium-Ion Magnetically Imprinted Materials

Compared to traditional imprinted polymers, lithium ion surface ion-imprinted polymers effectively overcome the challenge of imprinted sites being easily embedded. They significantly enhance mass transfer between recognition sites and lithium ions, thereby facilitating the elution and recombination of lithium ions [103]. However, the challenge of recycling imprinted materials persists. Consequently, numerous researchers have explored magnetic separation technology as a solution. For instance, magnetic carbon nanorods (Fe_3_O_4_), possessing magnetic properties, serve as the matrix carrier. When combined with surface ion imprinting technology, they enable the preparation of lithium ion magnetic-imprinted materials characterized by high specific selectivity and recyclability.

#### Preparation of Imprinted Magnetic Adsorbent Materials by Surface-Imprinted Polymerization Method

The preparation of magnetic lithium ion-imprinted polymers primarily employs surface ion-imprinting technology. This involves grafting a polymer layer with a lithium ion cavity structure onto the surface of magnetic carriers or in the nearby area [103], which is mainly divided into chemical grafting and sol-gel method, where the sol-gel method is mostly based on ethyl orthosilicate as the cross-linking agent.

**Figure 18:**
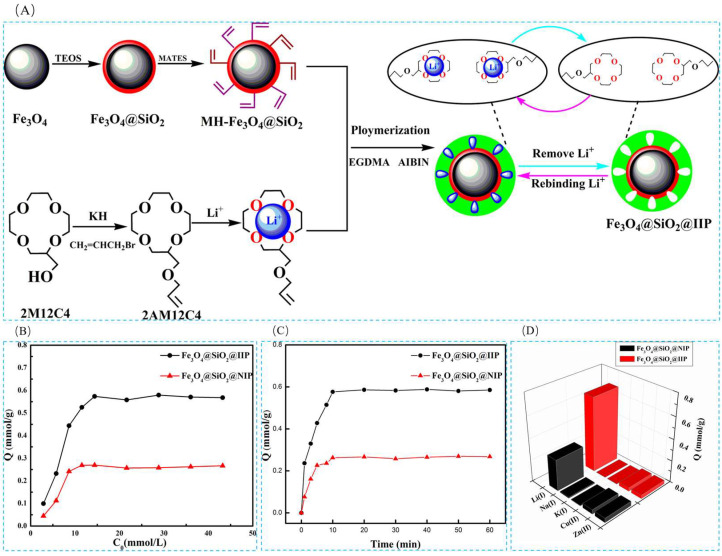
(**A**) Synthesis route of Fe_3_O_4_@SiO_2_@IIP (**B**) Li^+^ adsorption isotherms of Fe_3_O_4_@SiO_2_@IIP and Fe_3_O_4_@SiO_2_@NIP; (**C**) Li^+^ adsorption kinetics of Fe_3_O_4_@SiO_2_@IIP and Fe_3_O_4_@SiO_2_@NIP adsorption; (**D**) Li^+^ selective binding analysis of Fe_3_O_4_@SiO_2_@IIP and Fe_3_O_4_@SiO_2_@NIP selective binding analysis for Li^+^. Reproduced with permission from Ref. [38], *ACS Publications*, 2015.

Xubiao Luo et al. [38] synthesized magnetic lithium ion-imprinted polymers Fe_3_O_4_@SiO_2_@IIP with core-shell structure by surface imprint polymerization, using 2-(allyloxy)methyl-12crown-4 (2M12C4) as the functional monomer, LiCl·H_2_O as the template, EGDMA as the cross-linking agent, and AIBN as the initiator, with MH- Fe_3_O_4_@SiO_2_ magnetic matrix carrier, Fe_3_O_4_@SiO_2_@IIP was prepared, and Figure 18A shows its preparation flow. As shown in Figure 18B,C, the adsorption isotherms of this material indicate that the adsorbent has uniform adsorption sites, and the maximum adsorption capacity can reach 0.586 mmol·g^−1^. And as shown in Figure 18D, the Fe_3_O_4_@SiO_2_@IIP has excellent selectivity for Li^+^ in which the selective separation factors of Li^+^ with respect to Na^+^, K^+^, Cu^2+^, and Zn^2+^ are respectively 50.88, 42.38, 22.5, and 22.2.

**Figure 19:**
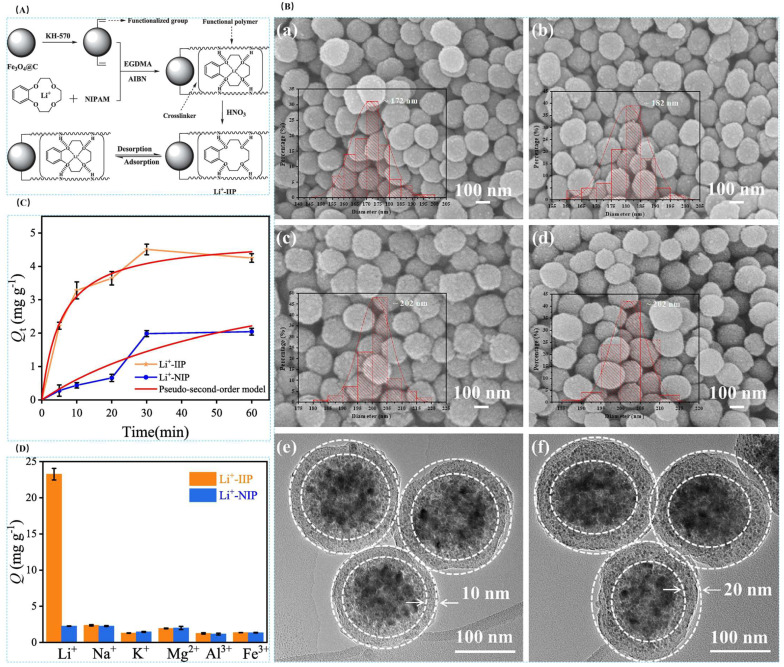
(**A**) Schematic of the synthesis route of Li^+^-IIP on Fe_3_O_4_@C surface, (**B**) FESEM and TEM images of (**a**,**e**) Fe_3_O_4_@C, (**b**) KH-570-modified Fe_3_O_4_@C, (**c**,**f**) Li^+^-IIP and (**d**) Li^+^-NIP, (**C**) Li^+^ adsorption on Li^+^-IIP and Li^+^-NIP kinetics, (**D**) selectivity study of Li^+^ on Li^+^-IIP. Reproduced with permission from Ref. [31], *Elsevier*, 2023.

Chang Liang et al. [31] prepared novel temperature and magnetic dual-responsive ion-imprinted materials by surface-imprinted polymerization, using LiClO_4_ as template ion, B12C4 as complexing agent, NIPAM as functional monomer, EGDMA as cross-linking agent, and AIBN as initiator, and silane coupling agent-modified Fe_3_O_4_@C as the carrier to obtain Fe_3_O_4_@C@IIP, and the process is shown in Figure 19A. The material reached a maximum adsorption value of 23.46 mg·g^−1^ at 25 °C and 60 min. As observed by FESEM and TEM, all the materials were spherical with the inner core wrapped by the outer layer to form a core-shell structure as can be seen in Figure 19B. The saturated adsorption capacity of Li^+^-IIP in the isothermal adsorption curve in Figure 19C was 10.2 times higher than that of Li^+^-NIP, up to 23.46 mg·g^−1^, and the selectivity of Li^+^-IIP for Li^+^ was better than that of Na^+^, K^+^, Mg^2+^, Al^3+^, and Fe^3+^ which all demonstrated the high efficiency adsorption of the material with good selectivity.

**Figure 20:**
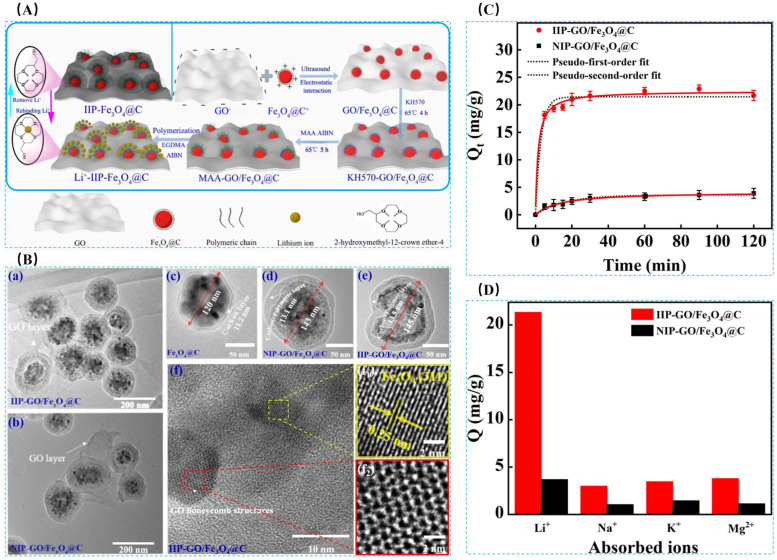
(**A**) Synthesis route of IIP-GO/Fe_3_O_4_@C, (**B**) TEM images of IIP-GO/Fe_3_O_4_@C (**a**) and NIP-GO/Fe_3_O_4_@C (**b**), NIP-GO/Fe_3_O_4_@C (**d**) and IIP-GO/Fe_3_O_4_@C (**e**) of Fe_3_O_4_@C (**c**), TEM photos of Fe_3_O_4_ nanoparticles, (**f**) enlarged TEM images of (**e**), (**f1**,**f2**) enlarged TEM images of (**f**), (**C**) adsorption kinetic curves of IIP-GO/Fe_3_O_4_@C and NIP-GO/Fe_3_O_4_@C, and (**D**) IIP-GO/Fe_3_O_4_@C and NIP-GO/Fe_3_O_4_@C adsorption selectivity. Reproduced with permission from Ref. [32], *Elsevier*, 2021.

Hong Zhao et al. [32] prepared a novel magnetic Li+ blotting composite IIP-GO/Fe_3_O_4_@C by surface-imprinted polymerization, using LiClO_4_ as the template ion, B12C4 as the functional monomer, DMF as the solvent, EGDMA as the cross-linking agent, AIBN as the initiator, and magnetic GO/Fe_3_O_4_@C as the composite support, and the preparation process is shown in Figure 20A. Figure 20B shows an SEM image of the composite, with the imprinted Fe_3_O_4_@C nanoparticles having a uniform particle size of about 145.2 nm, good inter-particle dispersion, and uniformly attached to the GO surface to form a hierarchical structure. In the isothermal adsorption diagram of Figure 20C, the maximum adsorption capacity of IIP-GO/Fe_3_O_4_@ for Li^+^ (31.24 mg·L^−1^) was higher than the maximum adsorption capacity of NIP-GO/Fe_3_O_4_@C (15.02 mg·L^−1^), and Figure 20D Schematic diagram of the adsorption selectivity factors of IIP-GO/Fe_3_O_4_@C and NIP-GO/Fe_3_O_4_@C materials show that the IIP materials have good adsorption capacity and selectivity.

Qi Liang et al. [104] also used the surface imprint polymerization method, using LiClO4 as the template ion, 2-hydroxymethyl-12-crown-4 as the complexing agent, MAA functional monomer, and EGDMA as the cross-linking agent, and modified magnetic carbon nanospheres, PMAA-Fe_3_O_4_@C, as the matrix carrier, to form an imprinted layer on the surface of PMAA-Fe_3_O_4_@C to prepare the PMAA- Fe_3_O_4_@C with a high selective adsorption capacity for Li^+^. Li^+^-IIP- Fe_3_O_4_@C with high selective adsorption capacity and its maximum adsorption capacity can reach 22.26 mg·g^−1^.

### 4.3. Lithium Ion-Imprinted Membrane Materials

The lithium-imprinted membrane’s large specific surface area facilitates the creation of more adsorption sites on its surface. This enhancement not only improves separation and recovery capabilities but also enhances adsorption capacity and selectivity. The preparation method is straightforward, often utilizing organic polymer materials like polyvinylidene fluoride and polyethersulfone as membrane materials. These materials offer advantages such as robust antioxidant activity, excellent chemical resistance, and strong thermal stability. Additionally, membrane performance can be further enhanced through surface grafting of functional groups [77].

#### 4.3.1. Preparation of Imprinted Adsorbent Membranes by Surface-Imprinted Polymerization Method

**Figure 21:**
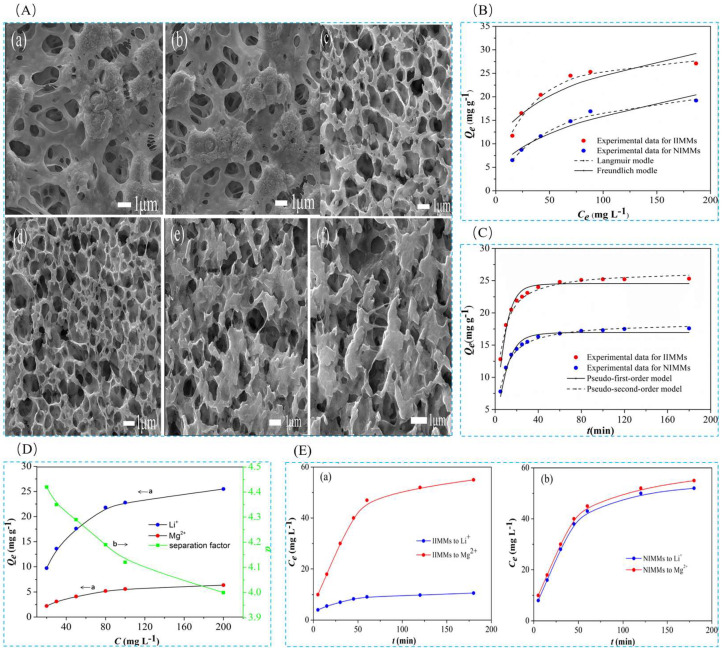
(**A**) SEM images of pristine PVDF macroporous membranes (**a**,**b**) pDA@PVDF macroporous membranes (**c**,**d**) and IIMMs (**e**,**f**); (**B**) adsorption isotherms and fitted models of Li^+^ on IIMMs and NIMMs; (**C**) adsorption kinetic curves and fitted models of Li^+^ on IIMMs and NIMMs; (**D**) adsorption selectivity (a) and separation factor (b) of IIMMs on Li^+^ and Mg^2+^ (**E**) selective permeability properties of IIMMs (**a**) and NIMMs (**b**) on Li^+^ and Mg^2+^. Reproduced with permission from Ref. [105], *Elsevier*, 2017.

Sun et al. [105] prepared a lithium ion-imprinted membrane IIMMs in 2017 in order to extract lithium resources from a salt lake, with the help of heterogeneous polydopamine (PDA) enabling stable cross-linking interactions between poly(vinylidene fluoride) (PVDF) macroporous membrane matrices to obtain a stabilized structure. The material was prepared by using LiCl as the template ion and 2-hydroxymethyl-12-crown ether-4 (2M12C4) as the functional monomer, EGDMA as the cross-linking agent, and AIBN as the initiator were used to prepare the IIMMs by surface-imprinted polymerization. The SEM images in Figure 21A show the evolution of the morphology of several types of macroporous membranes from PVDF to PDA@PVDF and finally to the IIMMs, and the adsorption isotherms and adsorption kinetics are characterized in Figure 21B,C for the adsorption of the material. The maximum adsorption capacity of Li^+^ was 19.2 mg·g^−1^, and the selective adsorption and permeation selectivity of this material for Li^+^ was excellent as shown in Figure 21D,E.

**Figure 22:**
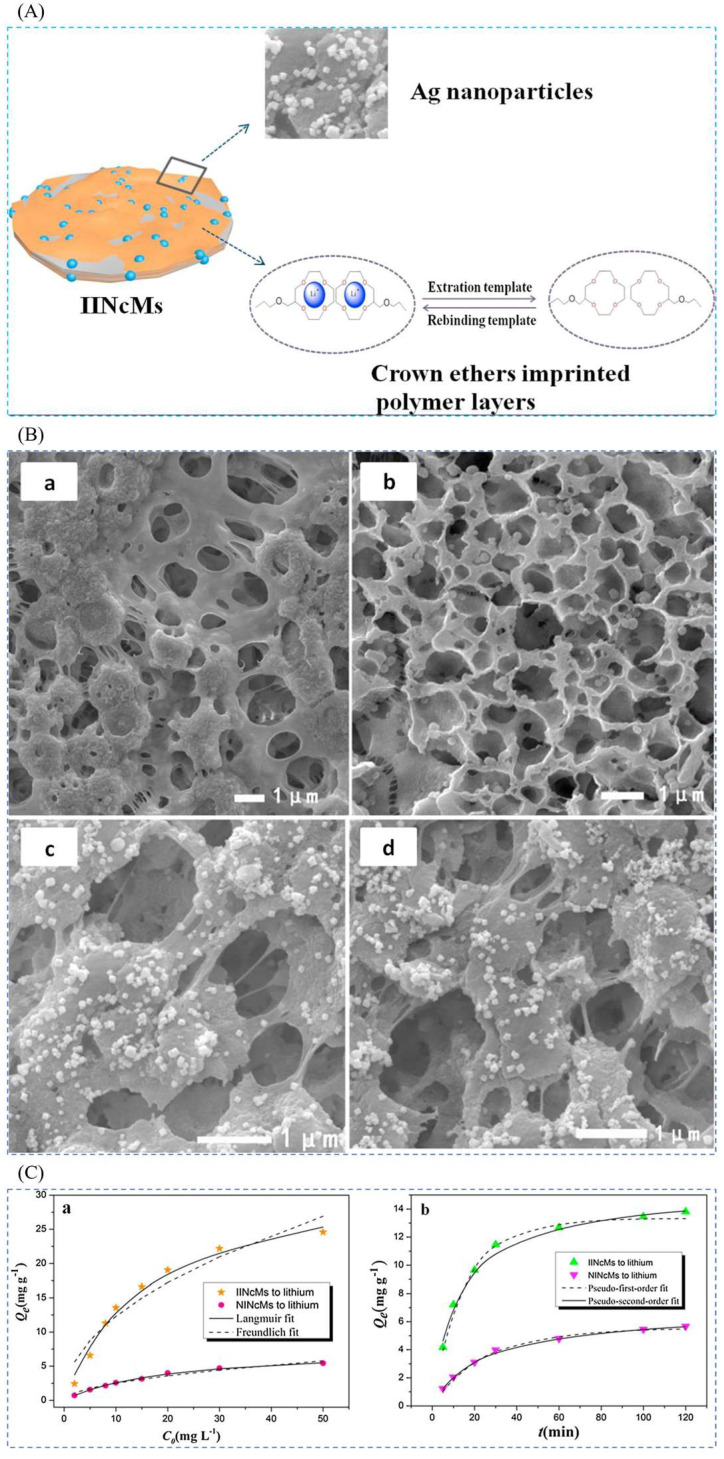
(**A**) Schematic structure of IINcMs; (**B**) (**a**) SEM images of pristine PVDF membranes, (**b**) PDA/PVDF membranes, (**c**) SEM image of Ag nanoparticles on the surface of Ag/PDA/PVDF membrane, (**d**) SEM image of uniformly imprinted layer on the surface of IINcMs; (**C**) (**a**) equilibrium data of lithium adsorption on IINcMs and NINcMs as well as Langmuir fitting and Freundlich fitting, (**b**) lithium adsorption on IINcMs and NINcMs with kinetic curves and fitted models. Reproduced with permission from Ref. [40], *Elsevier*, 2018.

Sun et al. [40] in 2018 obtained Ag/PDA/PVDF membranes after modification with silver (Ag) nanoparticles with the help of heterogeneous polydopamine (PDA), which were modified with 3-methacryloxypropyltrimethoxysilane (MPTS) to obtain a functional product (MPTS-Ag/PDA/PVDF), which was then prepared by surface-imprint polymerization method through which ion-imprinted nanocomposite membranes (IINCMs) were obtained. In this study, IINCMs were prepared by surface-imprinted polymerization on MPTS-Ag/PDA/PVDF membranes using LiCl as the template ion, 2M12C4 as the functional monomer, EGDMA as the cross-linking agent, and AIBN as the initiator. Porous ion-imprinted nanocomposite membranes with high hydrophilicity were prepared by the study, as can be seen in Figure 22A. The SEM images in Figure 22B show the morphological changes from PVDF to PDA/PVDF to Ag/PDA/PVDF and finally to IINCMs, and the distribution of the imprinted layers is very uniform as can be seen from analyzing the SEM images of IINCMs. Figure 22C shows the adsorption isotherm and adsorption kinetics characterization of the material, which has a maximum adsorption capacity of 25.58 mg·g^−1^ for Li^+^.

#### 4.3.2. Electrochemical Preparation of Imprinted Adsorbent Membranes

**Figure 23:**
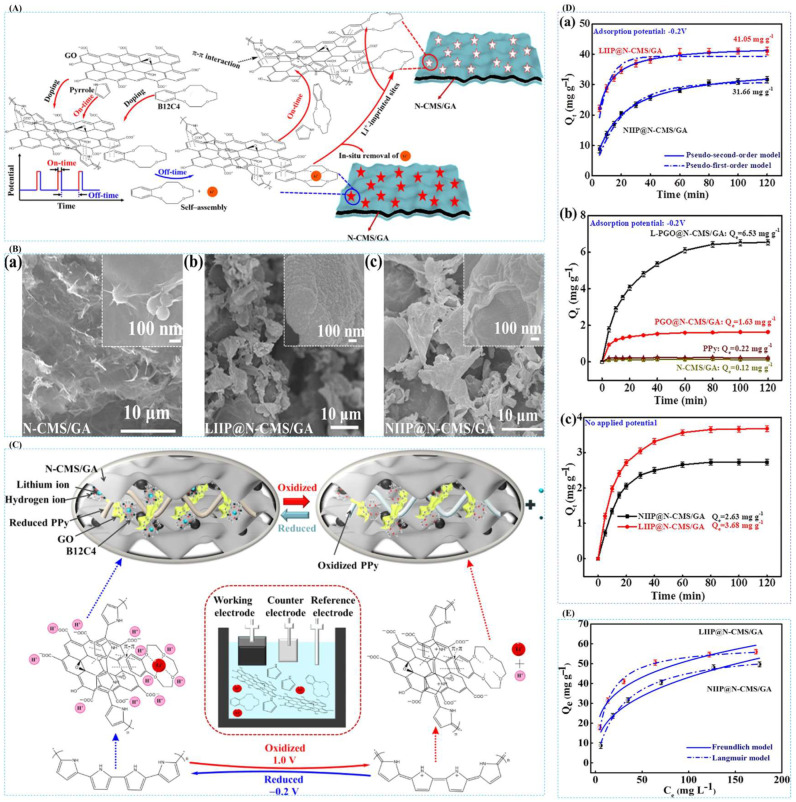
(**A**) Schematic diagram of the formation of LIIP@N-CMS/GA-imprinted film; (**B**) SEM images of (**a**) N-CMS/GA, (**b**) LIIP@N-CMS/GA, and (**c**) NIIP@N-CMS/GA; (**C**) electrochemical absorption–desorption mechanism of lithium ions on LIIP@N-CMS/GA electrodes; (**D**) simulated acidic leach solution of fly ash on the adsorption kinetic curves of Li^+^ on LIIP @N-CMS/GA and NIIP@N-CMS/GA electrodes with adsorption kinetic curves (**a**), L-PGO@N-CMS/GA, PGO@N-CMS/GA, PPy, and N-CMS/GA electrodes (**b**); and LIIP@N-CMS/GA and NIIP@N-CMS/GA with no applied potential (**c**); (**E**) nonlinear fitting curves for Langmuir model and Freundlich model adsorption isotherms. Reproduced with permission from Ref. [39], *Elsevier*, 2018.

Zhang et al. [39], in 2020, in order to extract lithium resources from an acidic system, successfully prepared ion-imprinted graphene-based hybridized aerogel (LIIP@N-CMS/GA). The formation process of LIIP@N-CMS/GA-imprinted film can be given a clearer overall perception from Figure 23A; the SEM images of Figure 23B show the surface morphology of N-CMS/GA, LIIP@N-CMS/GA, and NIIP@N-CMS/GA, respectively, and based on these morphology characterizations, it can be analyzed that the material has good adsorption properties; Figure 23C clearly shows the Li^+^ uptake and desorption mechanism; Figure 23D,E are the adsorption kinetics and adsorption isotherm characterization of this material, and the maximum adsorption capacity of this material for Li^+^ is 59.58 mg·g^−1^.

**Figure 24:**
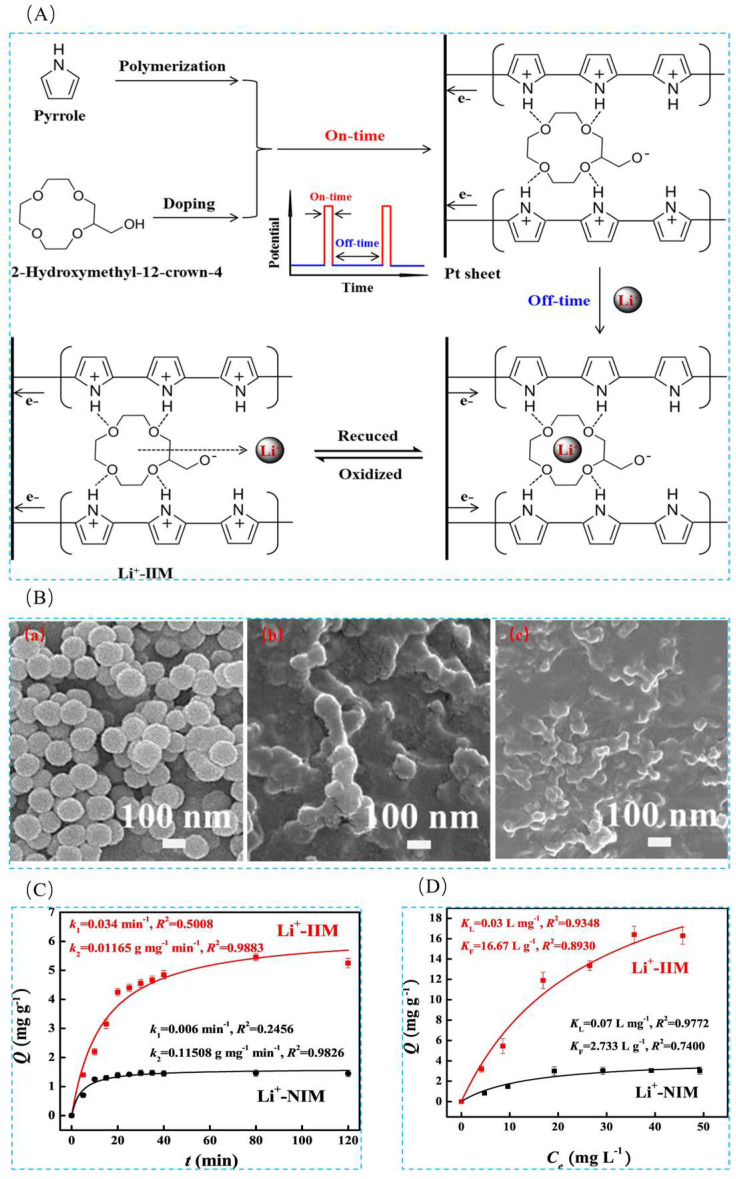
(**A**) Schematic of the synthesis of Li^+^-IIM membranes; (**B**) FESEM photographs of PPy nanospheres (**a**), Li^+^-NIM (**b**) and Li^+^-IIM (**c**); (**C**) effect of time on the adsorption capacity of Li^+^-IIM and Li^+^-NIM; (**D**) effect of initial Li^+^ concentration on the adsorption capacity of Li^+^-IIM and Li^+^-NIM effects. Reproduced with permission from Ref. [42], *Elsevier*, 2020.

In order to meet the major challenge of lithium extraction from acidic systems, Liu et al. [42], with the help of electrochemical switched ion exchange (ESIX) technology, combined with ion-imprinting technology, using unipolar pulsed electropolymerization method, LiCl as the template ion, 2AM12C4 as the functional monomer, and pyrrole as the conductive and cross-linking agent, successfully prepared lithium ion-imprinted membranes (Li^+^-IIM). A clearer overall perception of the study can be obtained from Figure 24A; the FESEM images in Figure 24B show the surface morphology of ppy nanorods, Li^+^-NIM, and Li^+^-IIM, respectively; Figure 24C,D are the adsorption kinetics and adsorption isotherm characterization of the material, which has a maximum adsorption capacity of 16.40 mg·g^−1^ for Li^+^.

#### 4.3.3. Preparation of Imprinted Adsorbent Membranes by Hydrolysis Polymerization

**Figure 25:**
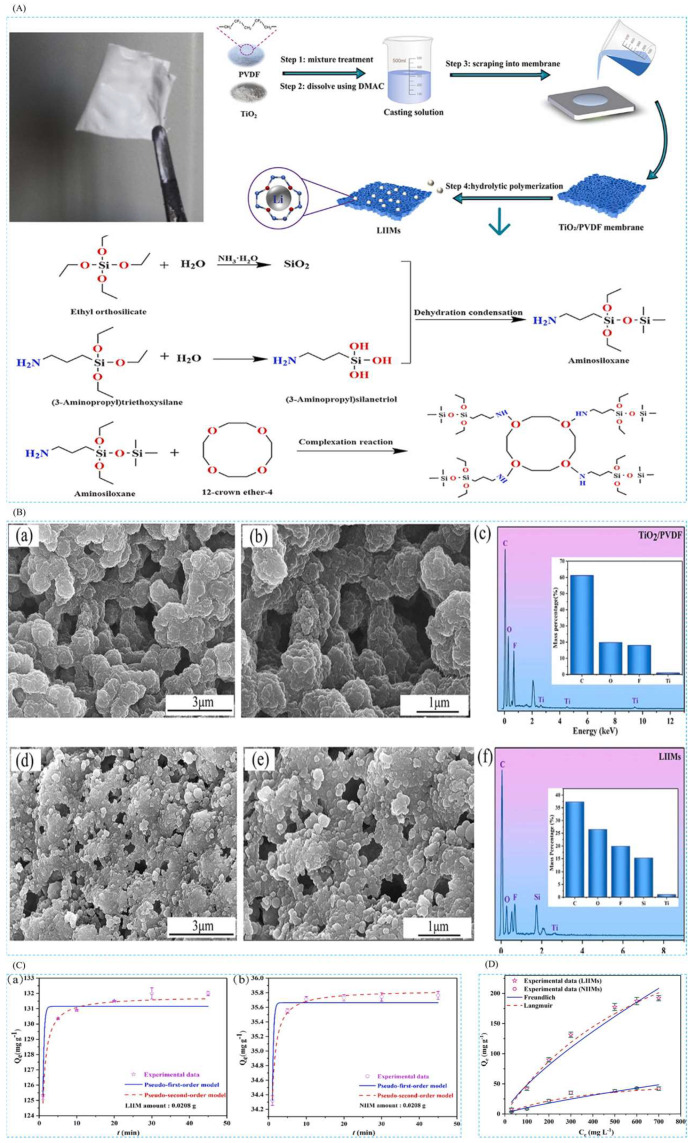
(**A**) Schematic diagram of the synthesis of LIIMs; (**B**) SEM photographs of TiO_2_/PVDF (**a**,**b**), LIIMs (**d**,**e**), and EDS spectra of TiO_2_/PVDF (**c**) and LIIMs (**f**); (**C**) adsorption kinetic curves for Li^+^ adsorption on LIIMs (**a**) and NIIMs (**b**) curves; (**D**) nonlinear fitting curves of Langmuir model and Freundlich model for Li^+^ adsorption on LIIMs and NIIMs. Reproduced with permission from Ref. [44], *Elsevier*, 2022.

Yang et al. [44], in 2022, in order to selectively recover lithium from spent lithium-ion batteries, successfully synthesized lithium ion-imprinted membranes (LIIMs) with very good adsorption properties by using hydrolysis polymerization combined with lithium ion-imprinting technology. In this study, LIIMs were prepared by using LiCl as the template ion, 12C4E as the complexing agent, KH550 (APTES) as the functional monomer, tetraethyl silicate (TEOS) as the cross-linking agent, and PVDF as the carrier. The synthesized materials and their synthesis are shown in Figure 25A, and the surface topography of the membrane can be clearly seen in the SEM image in Figure 25B, and the EDS image supports the successful preparation of the membrane; Figure 25C,D show the adsorption isotherms and adsorption kinetics characterization of the material, which has a maximum adsorption capacity of 132 mg·g^−1^ for Li^+^.

**Figure 26:**
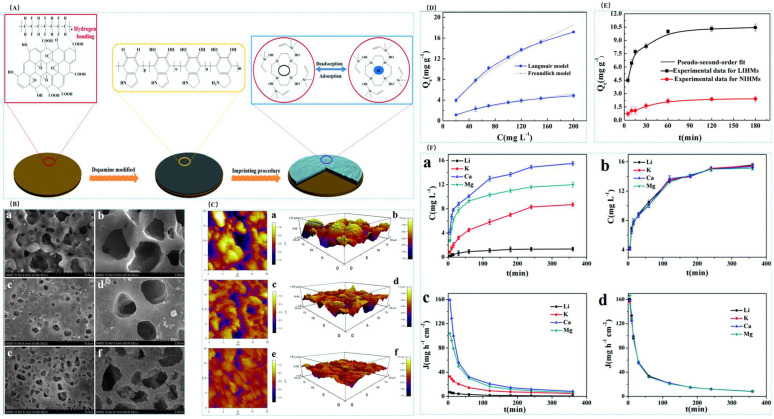
(**A**) Schematic of the preparation method of LIHMs with selective adsorption of Li^+^; (**B**) SEM photos of GO/PVDF (**a**,**b**), pDA@GO/PVDF (**c**,**d**) and LIHMs (**e**,**f**); (**C**) AFM images of GO/PVDF (**a**,**b**), pDA@GO/PVDF (**c**,**d**) and AFM images of LIHMs (**e**,**f**); (**D**) nonlinear fitting of Langmuir and Freundlich models; (**E**) nonlinear fitting of pseudo-second-order model; (**F**) permeability performance of LIHMs (**a**) and NIHMs (**b**), and LIHMs (**c**) and NIHMs (**d**) with time-dependent permeation flux curves. Reproduced with permission from Ref. [41], *RSC Publishing*, 2017.

Cui et al. [41] prepared antifouling lithium-imprinted hybrid membranes (LIHMs) by hydrolysis polymerization with the help of polydopamine (PDA) using PVDF/GO hybrid membranes as the base membranes in order to cope with the problem of ion-imprinted membrane contamination in 2018. In this study, LIHMs were obtained by polymerization reaction on pDA@GO/PVDF membranes using LiCl as template ion, 12C4E as complexing agent, vinyltriethoxysilane (VTES) as functional monomer, and TEOS as cross-linking agent. The preparation process is shown in Figure 26A; SEM of Figure 26B can clearly see that after the polydopamine modification, the surface of the pDA@PVDF/GO membrane formed a multilevel structure and the polymer network was deposited on the pDA@PVDF/GO membrane; the three AFM images of Figure 26C show the presence of polymer layers on the hybridized membrane; Figure 26D,E are the adsorption isotherms and adsorption kinetics characterization of the material, which show the maximum adsorption capacity of 27.10 mg·g^−1^ for Li^+^, and Figure 26F shows the high selectivity of this material for Li^+^.

### 4.4. Other Types of Lithium Ion-Imprinted Materials

Besides the materials mentioned earlier, alternative substances can extract lithium resources. Below, various methods for extracting lithium resources are outlined.

#### 4.4.1. Preparation of Imprinted Adsorbent Aerogels by Surface Imprint Polymerization

**Figure 27:**
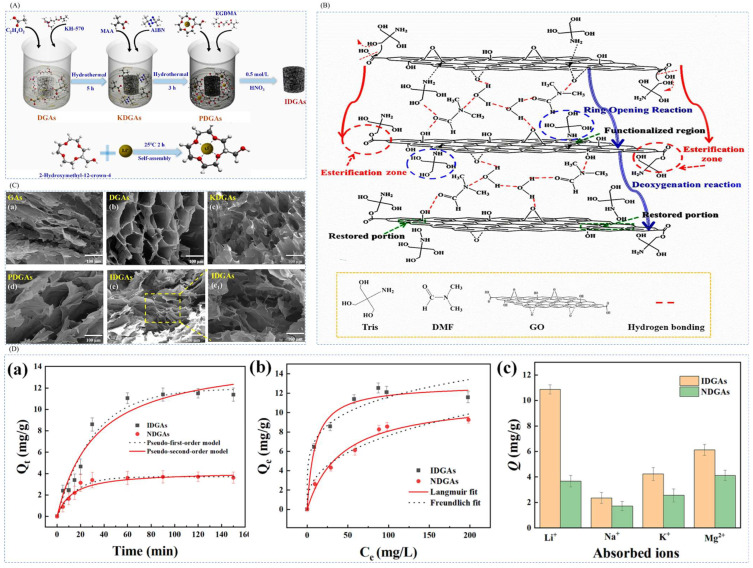
(**A**) Comprehensive roadmap of IDGAs; (**B**) mechanism of formation of DGAs; (**C**) FESEM images of (**a**) GAs, (**b**) DGAs, (**c**) KDGAs, (**d**) PDGAs, and (**e**,**e_1_**) IDGAs; (**D**) adsorption kinetic profiles of (**a**) IDGAs and NDGAs, (**b**) adsorption isothermal curves, (**c**) adsorption selectivity. Reproduced with permission from Ref. [45], *Elsevier*, 2024.

Kang et al. [45] used graphene oxide (GO) as the backbone material and tris(hydroxymethyl)aminomethane (Tris) as the first new method of reducing agent. Li^+^-imprinted three-dimensional embedded graphene aerogels (IDGAs) were successfully constructed by liquid-phase self-assembly technique combined with surface modification and assisted cross-linking by DMF. The graphene aerogels were characterized by structural stability, high selectivity, and high recyclability. The IDGAs not only prevented the accumulation of GO, but also introduced additional adsorption sites, which significantly improved the efficiency and capacity of Li^+^ adsorption. The IDGAs were prepared by using LiClO_4_ as the template ion, 2M12C4 as the complexing agent, MAA as the functional monomer, DGDMA as the cross-linking agent, and AIBN as the initiator. IDGAs were prepared by surface blotting method by adding DGA (Figure 27B shows the formation mechanism of DGAs) as a carrier as shown in Figure 27A. Figure 27C shows the FESEM images of GA, DGA, KDGA, PDGA, and IDGA); Figure 27D shows the adsorption kinetic curves of IDGAs and NDGAs at 25 °C. By comparison, the equilibrium adsorption capacity of the imprinted material IDGAs was 11.50 mg·g^−1^, which was much higher than that of the non-imprinted material NDGAs (3.71 mg·g^−1^).

#### 4.4.2. Functionalized Imprinted Polymer Brushes Prepared by UV-Initiated Surface Polymerization

**Figure 28:**
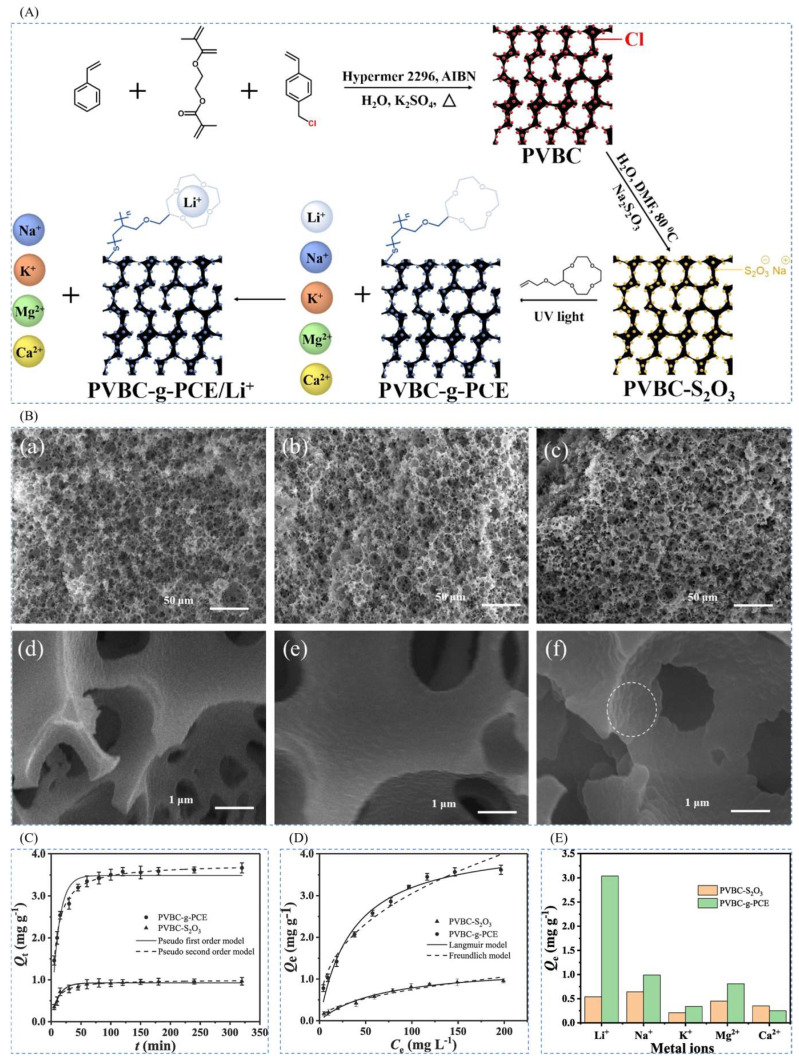
(**A**) Synthesis route of PVBC-g-PCE and its adsorption mechanism on Li^+^; (**B**) SEM photographs of PVBC (**a**), PVBC- S_2_O_3_ (**b**), PVBC-g-PCE (**c**), and expanded pore surface of PVBC (**d**), PVBC- S_2_O_3_ (**e**), and PVBC-g- PCE (**f**); (**C**) kinetic data and modeling of Li^+^ adsorption on PVBC- S_2_O_3_ and PVBC-g-PCE; (**D**) equilibrium data of Li^+^ adsorption on PVBC- S_2_O_3_ and PVBC-g-PCE; and (**E**) selective metal ion uptake by VBC- S_2_O_3_ and PVBC-g-PCE. Reproduced with permission from Ref. [46], *Elsevier*, 2020.

Xue et al. [46] synthesized macroporous high internal phase emulsion (polyHIPE) foam polymers by ultraviolet (UV)-initiated surface polymerization in 2020 for the purpose of recovering lithium-ion resources from brines of salt lakes, i.e., as a 2-(allyloxy)hydroxymethyl-12-crown-4 ether (2AM12C4)-functionalized polymer brush (PVBC-g-PCE). The chloromethyl-functionalized porous polymer (PVBC) was first modified in chloromethyl to become PVBC- S_2_O_3_, and the surface polymerization of 2AM12C4 with UV-initiated PVBC- S_2_O_3_ yielded PVBC-g-PCE, with LiCl as the ionic template, 2AM12C4 as the complexing agent, EGDMA as the cross-linking agent, AIBN as the initiator, and PVBC- S_2_O_3_ as the carrier to prepare the imprinted material, as shown in Figure 28A; Figure 28B shows the characterization of the material, and the success of the surface modification can be seen; Figure 28C,D show the adsorption kinetic data and the equilibrium data of adsorption of the material, respectively, and the maximal adsorption capacity of this material is 3.67 mg·g^−1^; Figure 28E shows the high selectivity to Li^+^.

#### 4.4.3. Preparation of Imprinted Nanofibers by Electrochemical Methods

**Figure 29:**
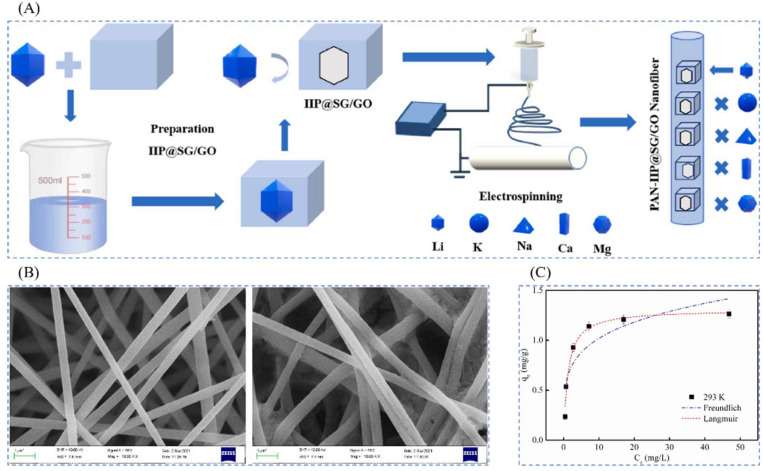
(**A**) Synthesis route of PAN-IIP@SG/GO nanofibers; (**B**) SEM photographs of PAN-IIP@SG/GO before and after adsorption of Li^+^; (**C**) comparison of Langmuir and Freundlich adsorption models for Li^+^ adsorption by adsorbent materials. Reproduced with permission from Ref. [47], *Elsevier*, 2022.

Ding et al. [47], in 2022, prepared a high-performance and low-cost adsorption of novel nanofibrous materials by combining electrostatic spinning technology with surface ion-imprinted in order to achieve the selective separation and recovery of Li^+^ from brines in salt lakes by first grafting N-(2-aminoethyl)-3-amino-propyltriethoxysilane (KH-791) onto chromatographic silica gel (SG), adding oxidized graphene (GO) assembly, partial modification of the double bond on the amino functional group SG/GO with maleic anhydride and glacial acetic acid, and modification of the phosphoryl group by adding vinylphosphonic acid. The ion-imprinted polymer IIP@SG/GO was obtained by using LiCl as the template ion, EGDMA as the cross-linking agent, AIBN as the initiator, and the SG/GO composite material as the carrier. Transparent and homogeneous spinning solutions were prepared from IIP@SG/GO and PAN fibers, and PAN-IIP@SG/GO nanofibers were obtained by high-pressure electrostatic spinning, and the synthesis route is as shown in Figure 29A; Figure 29B clearly shows the smooth fibers before adsorption and the embedded large particles on the fibers after adsorption; Figure 29C shows that the material adsorption reaches equilibrium within 120 min, and the maximum adsorption amount of Li^+^ is 1.1 mg·g^−1^. At the same time, the PAN-IIP@SG/GO has a higher selectivity for Li^+^.

### 4.5. Summary of Materials

Various methods have been developed to prepare different types of materials applicable in various environments for extracting lithium resources. These methods are summarized and compared in Table 2. For instance, the material Li/Rb-IHPS is an imprinted adsorption column. Its synthesis method is precipitation polymerization, with a template ion of LiCl-H2O, complexing agent 12C4, functional monomer MAA, cross-linking agent EGDMA, initiator AIBN, carrier HPS, and solvent CH3CN. The adsorption capacity of Li/Rb-IHPS is 2321.6 μg·g^−1^.

#### 4.5.1. Advantages and Disadvantages of Lithium Ion-Imprinted Adsorption Columns

Lithium ion-imprinted adsorption columns offer several advantages including high selectivity, efficiency, renewability, and environmental friendliness: (1) High selectivity: the lithium ion-imprinted adsorption column can be made highly selective by specific functional monomer design, which can efficiently capture and enrich lithium ions in aqueous solution while ignoring other metal ions. (2) High efficiency: due to its high selectivity, the lithium ion-imprinted adsorption column can enrich the target lithium ions from a large amount of aqueous solution into a small volume in a short time, and thus has a high enrichment efficiency and separation efficiency. (3) Regenerability: lithium ion-imprinted adsorption columns usually have a certain regenerability; by adjusting the solvent and conditions, the adsorbed lithium ions can be eluted, so that the adsorption columns can be reused, which reduces the cost. (4) Environmental friendliness: compared with traditional methods such as chemical precipitation, lithium ion-imprinted adsorption columns produce less waste liquid during lithium extraction, reducing the risk of environmental pollution.

Lithium ion-imprinted adsorption columns have the disadvantages of complex preparation, high cost, strict requirements for operating conditions, and limited market applications: (1) Complex preparation: the preparation of lithium ion-imprinted adsorption columns requires the synthesis of specific functional monomers and a series of polymerization, cross-linking, and post-processing steps; the preparation process is relatively complex, requiring a high level of technology and cost investment. (2) Higher cost: due to the complexity of the preparation process, the preparation cost of lithium ion-imprinted adsorption columns is higher, which may limit its promotion and application in large-scale industrial applications. (3) Strict operating conditions: in order to ensure the selectivity and efficiency of the lithium ion-imprinted adsorption columns, it is necessary to strictly control the operating conditions of the adsorption and elution process, including the selection of solvents, pH adjustment, etc., and the operation is relatively complex. (4) Restricted market application: at present, lithium ion-imprinted adsorption columns are mainly used in laboratory research and small-scale production, and have not been widely promoted in large-scale industrial applications, so the market application is restricted.

#### 4.5.2. Advantages and Disadvantages of Lithium Ion Magnetic-Imprinted Materials

Lithium ion magnetic adsorbent material has the advantages of high efficiency and rapidity, high selectivity, good controllability, renewability, and environmental friendliness: (1) high efficiency and rapidity: lithium ion magnetic adsorbent material has the ability to rapidly capture and enrich lithium ions, and the target ions can be rapidly enriched from the aqueous solution to a specific region by the applied magnetic field, which accelerates the separation and extraction process. (2) Strong selectivity: through rational design of material structure and functionalized surface, lithium ion magnetic adsorbent material can realize high selective adsorption of lithium ions, which can accurately identify and capture the target ions in the complex ion mixing system and improve the separation efficiency. (3) Good controllability: by adjusting the strength and direction of the applied magnetic field, the adsorption and release process of the lithium ion magnetic adsorbent material can be precisely controlled, making it highly controllable in operation. (4) Renewability: lithium ion magnetic adsorbent materials can usually be realized through simple heat treatment or solvent elution and other methods of ion release and material regeneration, which can reduce costs and reduce the consumption of resources. (5) Environmentally friendly: compared with traditional chemical methods, lithium ion magnetic adsorbent materials produce less waste liquid during lithium extraction, reducing the risk of environmental pollution.

Lithium ion magnetic adsorbent material has high preparation technology requirements, limited material stability, dependence on magnetic field, and in the research and development stage, has the following advantages: (1) high preparation technology requirements: lithium ion magnetic adsorbent material preparation technology is relatively complex; there is a need to synthesize specific functional materials and combine them with magnetic carriers, and the preparation process is relatively cumbersome, requiring a high level of technology and cost investment. (2) Limited material stability: some lithium ion magnetic adsorbent materials may be affected by chemicals in the aqueous solution or environmental conditions, leading to a decrease in adsorption performance or material failure, so it is necessary to strengthen the stability and durability management of the materials in the process of use. (3) Dependence on magnetic field: the adsorption and release process of lithium ion magnetic adsorbent material is affected by the applied magnetic field, so it needs to be equipped with magnetic field equipment, which increases the complexity of operation and the investment cost of equipment. (4) Still in the research and development stage: lithium ion magnetic adsorbent materials are still in the research and development stage in large-scale industrial applications, and are mainly used in laboratory research and small-scale production, and market applications are still subject to certain restrictions.

#### 4.5.3. Advantages and Disadvantages of Lithium Ion-Imprinted Adsorbent Membrane Materials

Lithium ion-imprinted adsorbent membrane materials have the advantages of high selectivity, fast response, good controllability, and resource saving: (1) High selectivity: lithium ion-imprinted adsorbent membrane materials can realize high selective capture of lithium ions through rational design of functional monomers and membrane structure, thus accurately identifying and enriching the target ions in the complex ion mixing solution, and improving the separation efficiency. (2) Fast response: due to its thin film structure, the lithium ion-imprinted adsorption membrane material is characterized by fast response, which can complete the adsorption and release process in a short time, accelerating the separation and extraction of lithium ions. (3) Good controllability: by adjusting the structure of the membrane and surface functionalization, the adsorption and release behavior of the lithium ion-imprinted adsorbent membrane material can be precisely controlled, making it highly controllable in operations. (4) Saving resources: compared with traditional chemical methods, lithium ion-imprinted adsorbent membrane materials produce less waste liquid during lithium extraction, reducing the risk of environmental pollution and saving water resources and treatment costs.

Lithium ion-imprinted adsorbent membrane materials have the disadvantages of high preparation technology requirements, limited material stability, dependence on operating conditions and are still in the research and development stage: (1) high preparation technology requirements: lithium ion-imprinted adsorbent membrane materials have a relatively complex preparation technology, which requires the synthesis of specific functional monomers and film-forming treatment on the membrane substrate, and the preparation process is relatively cumbersome, which requires a high technical level and cost investment. (2) Limited material stability: some of the lithium ion-imprinted adsorption membrane materials may be affected by chemicals in aqueous solution or environmental conditions, leading to a decline in adsorption performance or failure of the membrane material, so it is necessary to strengthen the management of the stability and durability of the material in the process of use. (3) Dependence on operating conditions: the adsorption and release processes of lithium ion-imprinted adsorbent membrane materials are affected by operating conditions, such as temperature, pH, etc., so the operating conditions need to be strictly controlled, which increases the complexity of operation. (4) Still in the research and development stage: lithium ion-imprinted adsorbent membrane materials in large-scale industrial applications are still in the research and development stage, and are mainly used in laboratory research and small-scale production, and market applications are still subject to certain restrictions.

## 5. Application of Lithium Ion-Imprinted Materials

The preparation of appropriate lithium ion-imprinted materials serves not only to recover Li^+^ but also to facilitate their application in various environments for lithium resource extraction. The following discussion will sequentially summarize the progress made in lithium ion-imprinted materials and briefly outline their applications in different contexts. This summary aims to provide insights into future directions for the development of lithium ion-imprinted materials.

### 5.1. Application of Lithium Ion-Imprinted Adsorption Columns

#### 5.1.1. Application of Precipitation Polymerization Method for the Preparation of Imprinted Adsorption Columns

**Figure 30:**
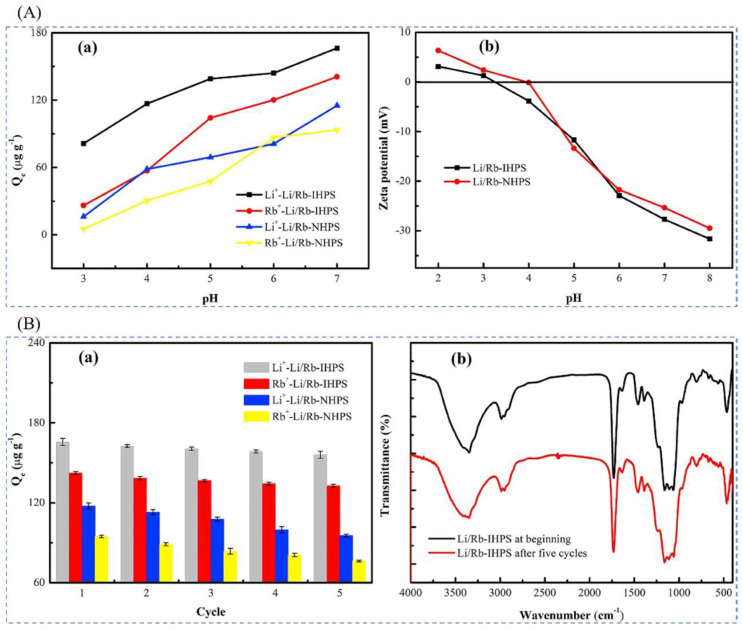
(**A**) Effect of pH on the adsorption capacity of Li^+^ and Rb^+^ by Li/Rb-IHPS and Li/Rb-NHPS (**a**) and the effect of pH on the zeta potentials of Li/Rb-IHPS and Li/Rb-NHPS (**b**); (**B**) (**a**) Li/Rb-IHPS and Li/Rb-NHPS reusability tests, (**b**) FT-IR spectra of Li/Rb-IHPS at the beginning and after five cycles. Reproduced with permission from Ref. [30], *Elsevier*, 2018.

Xu et al. [30] employed the precipitation polymerization method to create a novel Li/Rb imprinted multistage mesoporous silica (Li/Rb-IHPS). The study investigated the impact of pH on adsorption experiments. As shown in Figure 30A, the adsorption capacity of Li/Rb-IHPS for Li^+^ and Rb^+^ is pH-dependent. Specifically, the adsorption capacity of the Li^+^ adsorbent liquid gradually increases within the pH range of 3–7. Simultaneously, as the pH value increases from 2 to 8, the zeta potential gradually decreases, favorably enhancing the adsorption of Li^+^. In Figure 30B, the equilibrium adsorption capacity of Li/Rb-IHPS for Li^+^ and Rb^+^ remains consistently high at 94% and 93%, respectively, even after five cycles. This demonstrates the exceptional stability of Li/Rb-IHPS, making it a reliable choice for the repetitive extraction of Li^+^ and Rb^+^. However, Li/Rb-NHPS exhibits a lower equilibrium adsorption capacity, with values of 81% for Li^+^ and 80.5% for Rb^+^. This capacity is notably smaller compared to Li/Rb-IHPS, suggesting that Ion-Interaction Technology (IIT) effectively prevents the destruction of adsorption sites. Consequently, Li/Rb-IHPS emerges as a more suitable material for the recovery of lithium resources in neutral and low-concentration environments.

#### 5.1.2. Application of Imprinted Adsorption Columns Prepared by Surface Imprint Polymerization Method

**Figure 31:**
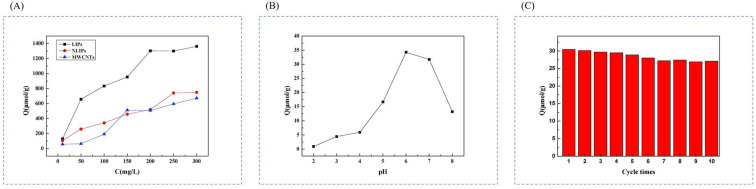
(**A**) Effect of initial concentration on Li^+^ adsorption capacity; (**B**) effect of pH on Li^+^ adsorption capacity; (**C**) adsorption–regeneration cycle of Li-IIPs. Reproduced with permission from Ref. [84], *Elsevier*, 2018.

Huang et al. [84] successfully synthesized lithium ion-imprinted polymers (Li^+^-IIP) on the surface of multi-walled carbon nanotubes using a surface-imprinted technique. Figure 31A demonstrates a proportional increase in adsorption capacity with rising initial concentrations (10 mg·L^−1^ to 200 mg·L^−1^). Based on these findings, the maximum adsorption capacities of Li^+^-IIPs and NLi^+^-IIPs were calculated to be 1362.56 μmol·g^−1^ and μmol·g^−1^, respectively. In Figure 31B, it can be seen that in the interval of pH = 2–6, the adsorption capacity of the material increased with the increase of pH value, and in the interval of pH = 6–8, with the increase of pH value, the adsorption capacity of the material started to decrease; the adsorption capacity of the material after 10 cycles of adsorption experiments only decreased by 10.3%, indicating that Li^+^-IIPs has good regeneration performance and has the potential of extracting and recovering Li^+^ from water. The optimal adsorption conditions for this material are a starting concentration of 200 mg·L^−1^, pH of 6, and temperature of 25 °C. Consequently, it can effectively extract lithium resources under conditions of higher concentration, weak acidity, and room temperature.

#### 5.1.3. Application of Electrochemically Prepared Imprinted Adsorption Columns

**Figure 32:**
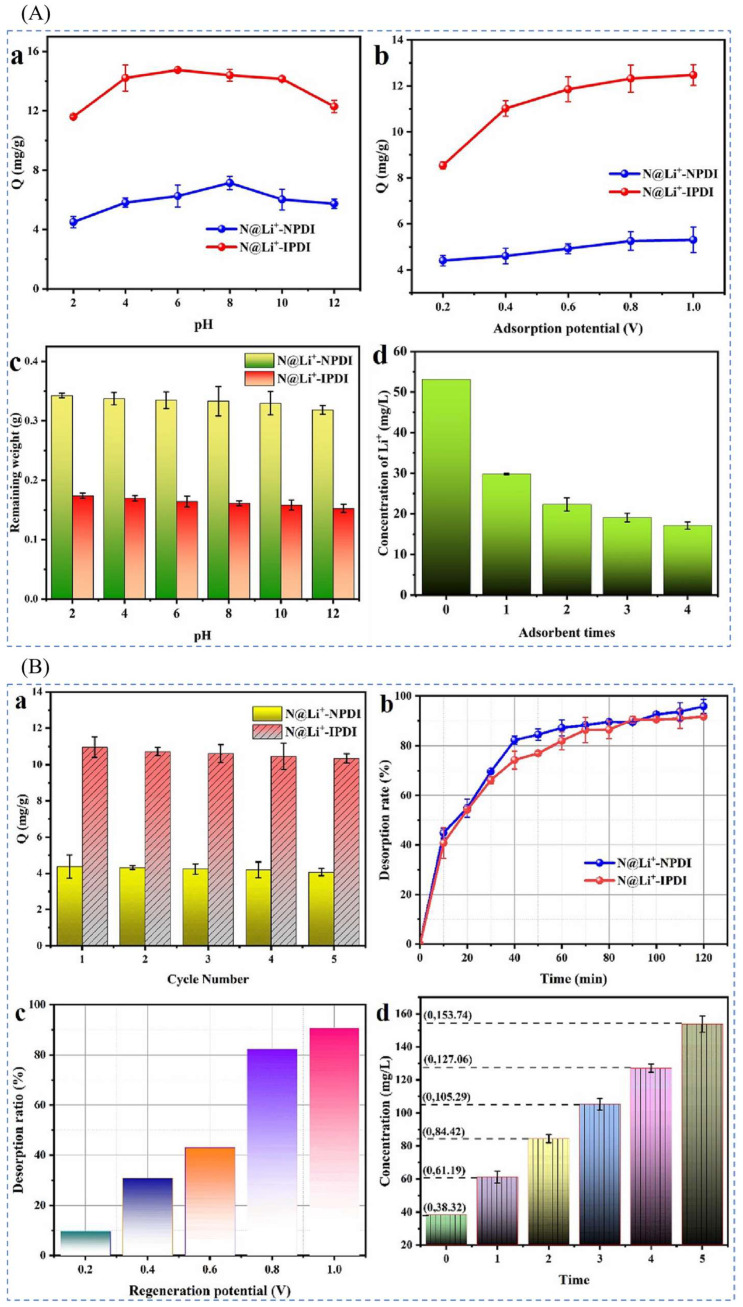
(**A**) (**a**) Adsorption with pH, (**b**) effect of voltage on adsorption capacity, (**c**) residual weight with pH, (**d**) Li^+^ concentration in solution with number of adsorptions; (**B**) (**a**) cyclic regeneration performance test of N@Li^+^-NPDI and N@Li^+^-IPDI electrodes; (**b**) N@Li^+^- NPDI and N@Li^+^-IPDI electrode materials; (**c**) desorption rate of N@Li^+^-IPDI electrode materials at different desorption potentials, and (**d**) relationship between the concentration of enriched solution and the number of desorption times. Reproduced with permission from Ref. [43], *Elsevier*, 2023.

Ning et al. [43] utilized an electrochemical method to synthesize the electrode material N@Li^+^-IPDI for selectively recovering lithium ions. Figure 32A illustrates the impact of pH, applied voltage, residual weight, and the number of adsorption cycles on the adsorption efficacy. The adsorption capacity escalates as the pH rises within the range of 2–6, with a peak recovery of 14.74 mg·g^−1^ at pH 6.0. Additionally, the adsorption efficiency correlates positively with increasing potential, albeit plateauing after reaching 0.6 V; the material loss showed a positive correlation with H^+^ concentration, resulting in a mass loss of 3.39% at pH = 2. After undergoing four cycles of resolution–adsorption, the Li^+^ concentration decreased from 53.1 mg·L^−1^ to 17.25 mg·L^−1^, with a recovery rate of 67.51%. Subsequently, after five regenerations, the material recovery reached 93.42% of the initial value. Desorption rates for the N@Li^+^-NPDI and N@Li^+^-IPDI electrodes were 95.82% and 91.72%, respectively. Notably, the N@Li^+^-IPDI exhibited consistent high regeneration rates across cycles. Furthermore, within the voltage range of 0.2–1.0 V, desorption rates increased proportionally with potential, the concentration of Li^+^ in the enrichment solution reached 153.74 mg·g^−1^.

### 5.2. Application of Lithium Ion Magnetic-Imprinted Materials

The unique magnetic properties of lithium ion magnetic-imprinted materials facilitate easier recycling. Additionally, the teams conducted studies on the materials’ adsorption properties under various conditions including temperature, pH value, and regeneration performance. This research aimed to determine the optimal application environment and effectiveness of these materials.

**Figure 33:**
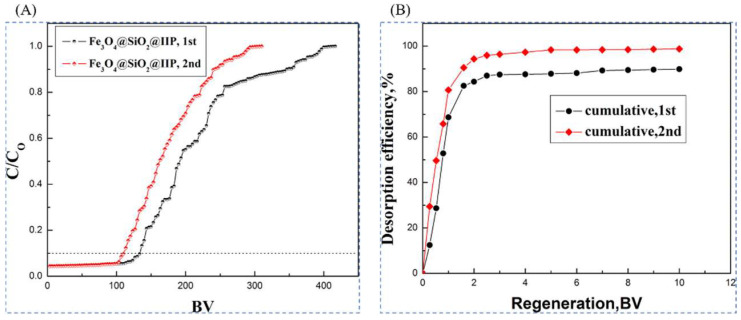
(**A**) Fixed-bed column adsorption test; (**B**) fixed-bed column regeneration test. Reproduced with permission from Ref. [38], *ACS Publications*, 2015.

Xubiao Luo et al. [38] investigated the effect of pH on the adsorption test, the adsorption capacity of Fe_3_O_4_@SiO_2_@IIP and Fe_3_O_4_@SiO_2_@NIP for Li^+^ at different pH (1–9), and the results showed that Fe_3_O_4_@SiO_2_@IIP can be used in a wide pH environment, and the pH of the solution is most suitable for 6.0. The research team also conducted a fixed-bed column adsorption test and a fixed-bed column regeneration test to verify the applicability of Fe_3_O_4_@SiO_2_@IIP for removing Li^+^ ions from wastewater. In Figure 33A, the fixed-bed column adsorption test shows the penetration curve of Fe_3_O_4_@SiO_2_@IIP toward Li^+^ ions, indicating that Fe_3_O_4_@SiO_2_@IIP exhibits stable Li^+^ ion removal performance. In Figure 33B, the fixed bed column regeneration test shows a cumulative desorption efficiency of about 89.9% for the first run and about 98.7% for the second run. This Fe_3_O_4_@SiO_2_@IIP is a material with high selectivity for lithium ions and can be efficiently recycled.

**Figure 34:**
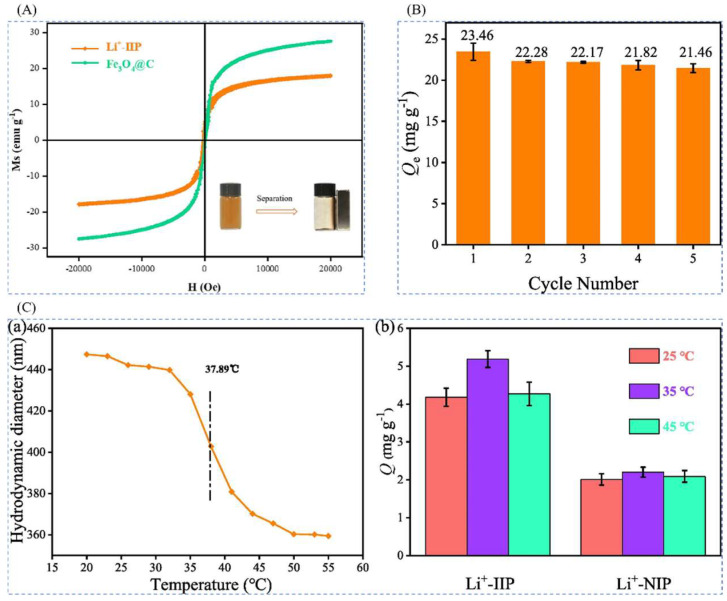
(**A**) The inset at the lower right corner shows the magnetization curves of Fe_3_O_4_@C and Li^+^-IIP, and the magnetic separation process of Li^+^-IIP; (**B**) cyclic adsorption properties of Li^+^-IIP; and (**C**) (**a**) hydrodynamic diameters and (**b**) adsorption capacities of Li^+^-IIP at different temperatures. Reproduced with permission from Ref. [31], *Elsevier*, 2023.

Chang Liang et al. [31] analyzed the magnetization curves of Fe_3_O_4_@C and Li^+^-IIP, depicted in Figure 34A. Additionally, they conducted five adsorption–desorption cycles on Li^+^-IIP particles, with the results presented in Figure 34B. Remarkably, even after these cycles, the equilibrium adsorption capacity remained at 91.47% of the initial value, affirming the cyclic stability of Li^+^-IIP. Furthermore, they verified the thermosensitive transition of Li^+^-IIP by conducting hydrodynamic diameter measurements in the in-liquid system, detailed in Figure 34C(a). Notably, the investigation also revealed the optimal adsorption temperature of the material to be 35 °C, as shown in Figure 34C(b). In conclusion, the Li^+^-IIP material offers effective temperature-switching adsorption of Li^+^ ions, coupled with reliable cyclic regeneration. Its magnetic properties also facilitate convenient recycling.

**Figure 35:**
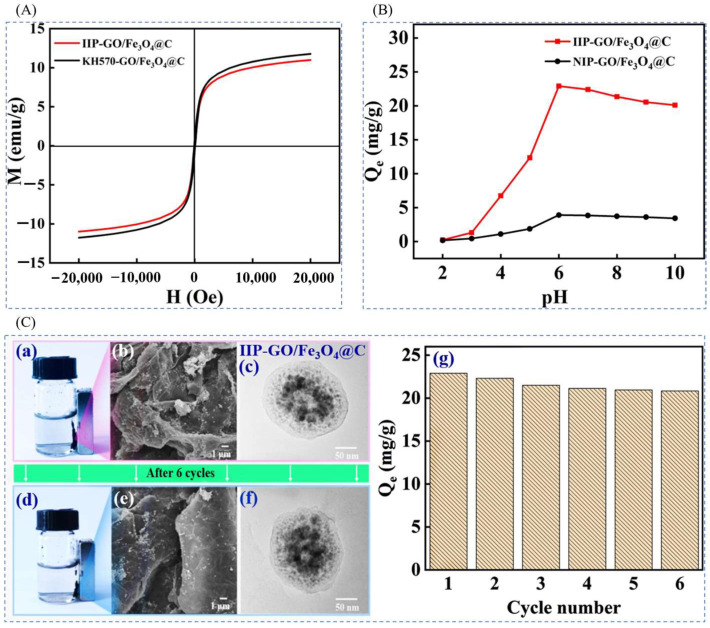
(**A**) Hysteresis lines of KH570-GO/Fe_3_O_4_@C and IIP-GO/Fe_3_O_4_@C; (**B**) effect of pH on the Li^+^ adsorption capacity of IIP-GO/Fe_3_O_4_@C and NIP-GO/Fe_3_O_4_@C; (**C**) adsorption target maps of IIP-GO/Fe_3_O_4_@C in six adsorption–desorption cycles (**a**,**d**), SEM maps (**b**,**e**) and TEM maps (**c**,**f**) of the regeneration performance of IIP-GO/Fe_3_O_4_@C (**g**). Reproduced with permission from Ref. [32], *Elsevier*, 2021.

Hong Zhao et al. [32] analyzed the hysteresis loops of KH570-GO/Fe_3_O_4_@C and IIP-GO/Fe_3_O_4_@C, illustrated in Figure 35A. They also explored the pH’s impact on the adsorption process, depicted in Figure 35B. The adsorption of Li^+^ on the IIP-GO/Fe_3_O_4_@C-imprinted material increased with rising pH, reaching a maximum adsorption capacity of 22.9 mg·g^−1^ at a pH of 6. The material’s regeneration performance was investigated. According to Figure 35C(g), the adsorption capacity of IIP-GO Fe_3_O_4_@C for Li^+^ decreased from 22.9 to 20.84 mg·g^−1^ after six adsorption–desorption cycles, representing a modest 9% reduction from the initial adsorption capacity. This finding highlights the commendable regeneration performance of IIP-GO/Fe_3_O_4_@C, indicating its potential for reuse.

### 5.3. Application of Lithium Ion-Imprinted Membrane Materials

#### 5.3.1. Application of Surface Imprint Polymerization for the Preparation of Imprinted Adsorbent Films

**Figure 36:**
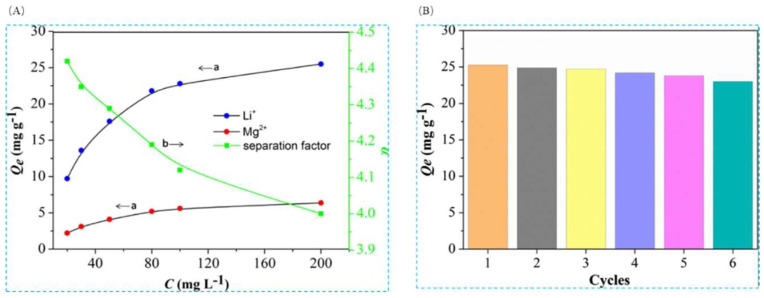
(**A**) Adsorption selectivity (a) and separation factors (b) of IIMMs for Li^+^ and Mg^2+^; (**B**) adsorption stability and regeneration performance of IIMMs after several adsorption–desorption cycles. Reproduced with permission from Ref. [105], *Elsevier*, 2017.

Sun et al. [105] utilized surface-imprinted polymerization to craft large porous polymer ion-imprinted microporous membranes (IIMMs) known for their robust renewability and heightened selectivity toward lithium ions. In this investigation, the adsorption selectivity of this material amid competing ions, such as Mg^2+^, was scrutinized. Figure 36A illustrates a distinct elevation in Li^+^ adsorption by the IIMMs in the presence of Mg^2+^, signifying enhanced specific recognition and adsorption selectivity for Li; the figure clearly shows that the maximum a-value (the selectivity factor) in the selective adsorption experiment was 4.42, indicating that IIMMs can maintain strong adsorption selectivity even when competing ions are present. Additionally, Figure 36B reveals that after six adsorption–elution cycles, the minimum adsorption amount decreased by only 9.09% compared to the maximum adsorption amount, demonstrating the material’s excellent regeneration performance.

**Figure 37:**
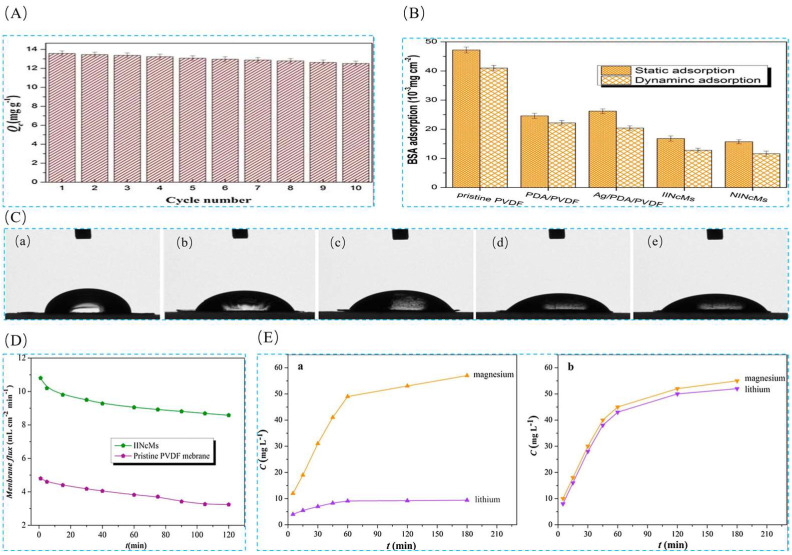
(**A**) Effect of regeneration performance of IINcMs on recombination capacity; (**B**) adsorption performance of BSA on membranes under static and dynamic conditions; (**C**) contact angles of (**a**) pure PVDF membranes, (**b**) PDA/PVDF, (**c**) Ag/PDA/PVDF, (**d**) IINcMs, and (**e**) NIINcMs; (**D**) contact angles of pure PVDF membranes and membrane fluxes of IINcMs; (**E**) time-dependent permeation selectivity curves of lithium and magnesium through (**a**) IINcMs and (**b**) NIINcMs. Reproduced with permission from Ref. [40], *Elsevier*, 2018.

Sun et al. [40] prepared ion-imprinted nanocomposite membranes (IINcMs) with high selectivity, and developed ion-imprinted nanocomposite membranes (IINcMs) through surface-imprinted polymerization, exhibiting high selectivity, regeneration, and antifouling properties. Figure 37A illustrates that after 10 adsorption–elution cycles, the minimum adsorption amount decreased by only 7.9% compared to the maximum adsorption amount, indicating excellent regeneration performance. To assess the antifouling performance of the prepared membrane, static and dynamic BSA adsorption experiments were conducted. Figure 37B demonstrates the strong antifouling performance of the resultant material. Additionally, the material exhibits strong hydrophilicity, evident from the contact angle measurement experiment depicted in Figure 37C, and membrane flux results shown in Figure 37D. Furthermore, the material demonstrates high selectivity, as illustrated in Figure 37E.

#### 5.3.2. Application of Electrochemically Prepared Imprinted Adsorbent Membranes

**Figure 38:**
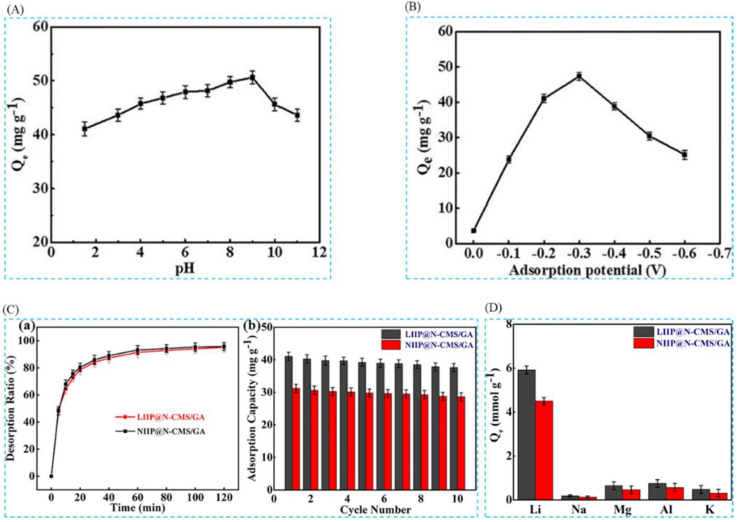
(**A**) Effect of pH on the adsorption capacity of Li^+^ on LIIP@N-CMS/GA electrode; (**B**) variation of adsorption capacity of Li^+^ on LIIP@N-CMS/GA electrode with adsorption potential by simulated acidic leach solution of fly ash; (**C**) desorption ratio curves of LIIP@N-CMS/GA and NIIP@N-CMS/GA electrodes (**a**); adsorption stability and regeneration performance of LIIP@N-CMS/GA and NIIP@N-CMS/GA electrodes (**b**); (**D**) adsorption capacity of fly ash simulated acidic leach solution on ions on LIIP@N-CMS/GA and NIIP@N-CMS/GA electrodes. Reproduced with permission from Ref. [39], *Elsevier*, 2018.

Zhang et al. [39] utilized a unipolar pulsed electropolymerization method to create a lithium ion-imprinted membrane (LIIP@N-CMS/GA) exhibiting high acid resistance. Figure 38A indicates that the membrane material achieved maximum adsorption capacity at pH = 9, with effective adsorption observed in both acidic and alkaline systems. To address practical application challenges, the study simulated the acidic leaching solution of fly ash (pH = 1.5) and explored the impact of various adsorption potentials on its efficacy. In Figure 38B, the maximum Li^+^ adsorption on the LIIP@N-CMS/GA electrode is observed at −0.3 V, reaching 47.31 mg·g^−1^. This adsorption process is further illustrated in Figure 38C, where the adsorption capacity of the LIIP@N-CMS/GA membrane remains at 91.7% of its initial value after 10 adsorption–elution cycling experiments. This resilience suggests the material’s promising recycling performance, making it highly suitable for large-scale lithium extraction. Figure 38D vividly demonstrates the material’s outstanding specific adsorption of Li^+^.

**Figure 39:**
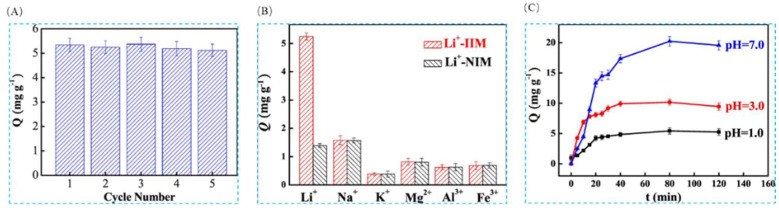
(**A**) Regeneration of Li^+^-IIM; (**B**) adsorption selectivity of Li^+^-IIM and Li^+^-NIM for Li^+^, Na^+^, K^+^, Mg^2+^, Al^3+^, and Fe^3+^; (**C**) kinetic adsorption curves of Li^+^-IIM at different pH values. Reproduced with permission from Ref. [42], *Elsevier*, 2020.

Liu et al. [42] employed unipolar pulsed electropolymerization to fabricate lithium ion-imprinted membranes (Li^+^-IIM) with high acid resistance. Figure 39A illustrates that even after five adsorption–elution cycling experiments, the Li^+^-IIM membrane retains 95.88% of its initial adsorption capacity. This suggests structural stability and efficient recycling performance, rendering it applicable in practical scenarios. Figure 39B illustrates the Li^+^-IIM membrane’s specific adsorption and selective recognition of the target ion Li^+^. Kinetic adsorption experiments were conducted on the Li^+^-IIM membrane at varying pH levels. The adsorption capacity of this membrane in acidic solutions, as depicted in Figure 39C, was lower than in neutral solutions. However, in contrast, the Li^+^-IIM membrane exhibited a stronger adsorption capacity in acidic conditions.

#### 5.3.3. Preparation of Imprinted Adsorbent Films using Hydrolysis Polymerization for Applications

**Figure 40:**
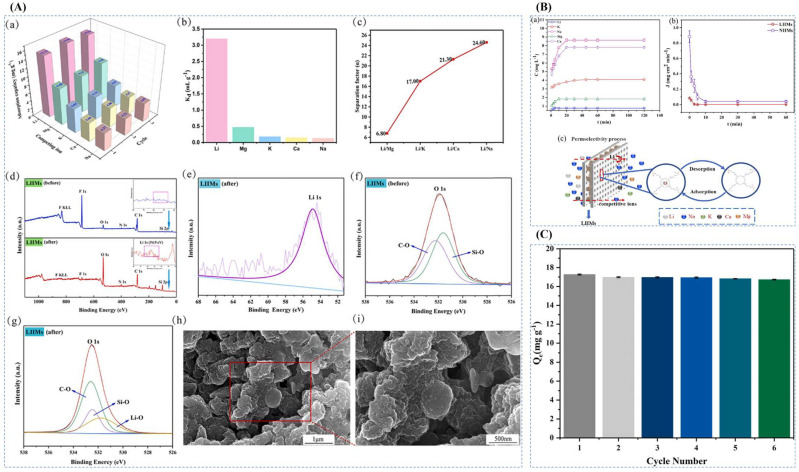
(**A**) Selective adsorption (**a**), ion distribution coefficients (**b**), and separation factors (**c**) of lithium ions by LIIMs of 40 mg·g^−1^ Li^+^/Mg^2+^, Li^+^/K^+^, Li^+^/Na^+^, and Li^+^/Ca^2+^; XPS full-scan spectra before and after adsorption of LIIMs (**d**) and Li (**e**) and O (**f**,**g**) high-resolution spectra and SEM after Li adsorption (**h**,**i**); (**B**) permeation capacity of LIIMs (**a**), permeation flux curves of LIIMs and NIIMs to Li^+^ (**b**), and schematic diagram of the permeation mechanism (**c**) (100 mg·g^−1^ of mixed solution of L^i+^, Na^+^, K^+^, Mg^2+^, and Ca^2+^); (**C**) adsorption stability results for LIIMs (40 mg·g^−1^ LiCl solution). Reproduced with permission from Ref. [44], *Elsevier*, 2022.

Yang et al. [44] synthesized lithium ion-imprinted membranes (LIIMs) via hydrolysis polymerization, demonstrating robust adsorption and selective recognition of Li^+^. Analysis of Figure 40A,B, along with SEM validation, reveals the membrane’s efficacy in complex solutions containing Li^+^ and four competing metal ions. Moreover, Figure 40C illustrates that, after six adsorption–elution cycles, the minimum adsorption amount decreased by only 3% compared to the maximum adsorption amount, indicating excellent regeneration performance of the material.

**Figure 41:**
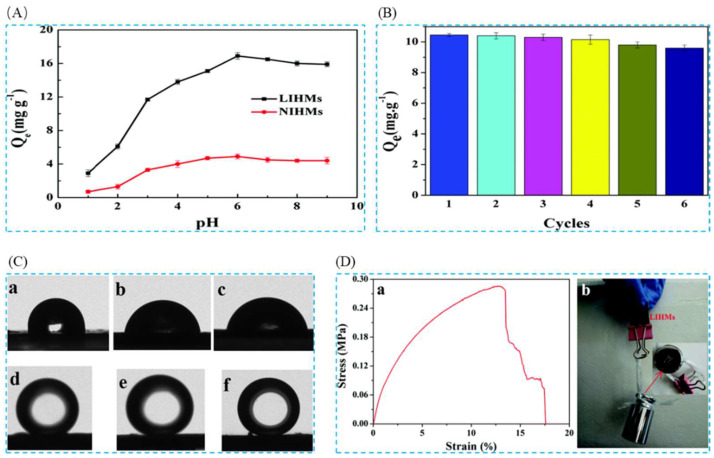
(**A**) Effect of pH on the adsorption capacity of LIHMs and NIHMs; (**B**) adsorption stability and regeneration performance of LIHMs; (**C**) water contact angle of PVDF (**a**), PVDF/GO (**b**), and LIHMs (**c**), underwater oil of PVDF (**d**), PVDF/GO (**e**), and LIHMs (**f**) contact angles; (**D**) (**a**) stress–strain curves of LIHMs, (**b**) pictures of LIHMs holding a 100 g weight. Reproduced with permission from Ref. [41], *RSC Publications*, 2017.

Cui et al. [41] synthesized antifouling lithium-imprinted hybrid membranes (LIHMs) via hydrolysis polymerization, showcasing robust selective recognition of Li^+^, excellent antifouling properties, and regeneration ability. The material’s adsorption capacity for Li^+^ significantly rises with increasing pH, peaking at pH=6, indicating its suitability for lithium resource recovery in neutral and weakly acidic environments. After six adsorption–elution cycles, the minimum adsorption capacity is only 8.2% lower than the maximum, demonstrating the material’s strong regeneration performance. In this experiment, the material’s antifouling performance was assessed through surface wettability, as depicted in Figure 41C. The results of the anti-pollution test indicate that the surface of the produced LIHMs formed a water layer, aiding in the prevention of organic contamination. Mechanical strength is crucial for evaluating membrane viability during separation. Figure 41D illustrates the tensile strength and Young’s modulus of the LIHMs, measuring 0.286 MPa and 5.151 MPa, respectively. Additionally, a single piece of LIHMs can support a weight of 100 g. In conclusion, the material exhibits good mechanical strength.

### 5.4. Other Types of Materials for Lithium Ion

#### 5.4.1. Application of Surface-Imprinted Polymerization for the Preparation of Imprinted Adsorbent Aerogels

**Figure 42:**
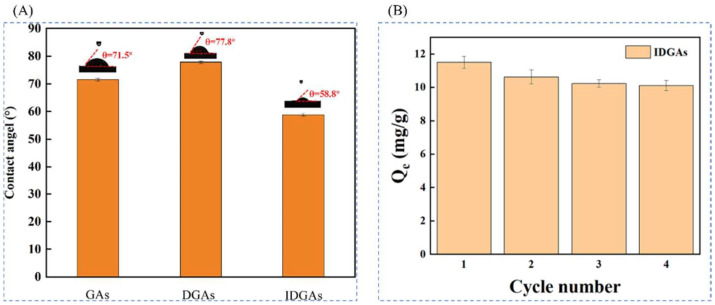
(**A**) Contact angles of GA, DGA, and IDGA; (**B**) regeneration performance. Reproduced with permission from Ref. [45], *Elsevier*, 2024.

Kang et al. [45] successfully created Li^+^-imprinted 3D block graphene aerogels (IDGAs) using graphene oxide (GO) as the backbone material and tris(hydroxymethyl)aminomethane (Tris). This was achieved through surface modification employing a liquid-phase self-assembly technique with cross-linking facilitated by DMF. Figure 42A illustrates the water contact angle test results for GAs, DGAs, and IDGAs, showcasing their exceptional wettability and stability. In Figure 42B, the shift in the adsorption value of IDGAs for Li is evident after four adsorption–desorption cycles. The adsorption amount decreased from 11.50 mg·g^−1^ to 10.11 mg·g^−1^, representing a 12% reduction from the initial value.

#### 5.4.2. Application of Functionalized Imprinted Polymer Brushes Prepared by UV-Initiated Surface Polymerization

**Figure 43:**
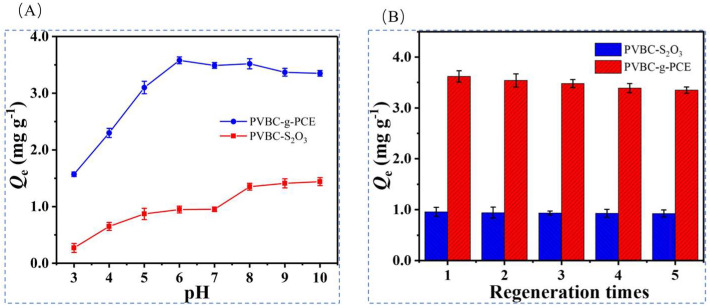
(**A**) Effect of pH on the adsorption capacity of PVBC-S_2_O_3_ and PVBC-g-PCE; (**B**) regeneration capability of PVBC-S_2_O_3_ and PVBC-g-PCE. Reproduced with permission from Ref. [46], *Elsevier*, 2020].

Xue et al. [46] synthesized macroporous polyHIPE foam polymers via UV-initiated surface polymerization. Figure 43A demonstrates the pH’s impact on adsorption performance. Adsorption capacity increases from pH 3 to 6, stabilizing as pH shifts from 6 to 10. Maximum performance occurs at pH 6.0. In Figure 43B, after five cycles of adsorption–desorption experiments, the material retains 96.4% of its initial adsorption capacity.

#### 5.4.3. Application of Electrochemical Methods for the Preparation of Nanofiber-Imprinted Materials

**Figure 44:**
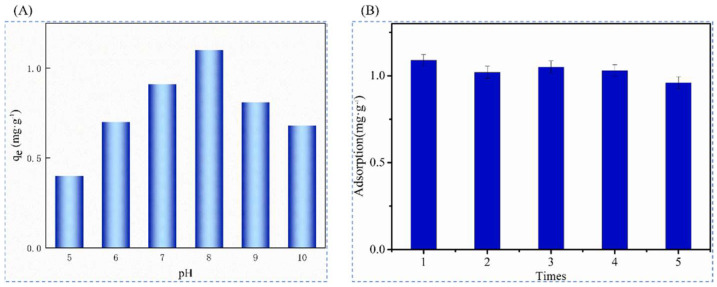
(**A**) Adsorption capacity of PAN-IIP@SG/GO for Li^+^ in the pH range of 5–10; (**B**) recovery performance. Reproduced with permission from Ref. [47], *Elsevier*, 2022.

Ding et al. [47] employed a combination of electrostatic spinning and surface ion-imprinted techniques to fabricate a novel nanofiber adsorption material, IIP@SG/GO. Figure 44A illustrates the pH effect on adsorption performance. Notably, at pH = 8.0, the recovery reached 1.1 mg·g^−1^. Additionally, Figure 44B demonstrates that the adsorption capacity of PAN-IIP@SG/GO only decreased by 10.91% after five cycles of adsorption–desorption experiments.

### 5.5. Application Summary

There are many types of lithium ion-imprinted materials, and they can be used in different environments for lithium extraction. The following Table 3 compares their adsorption capacity ranges, optimal pH and regeneration performance parameters, for example, the adsorption capacity of Li/Rb-IHPS in the experimental range of 0–200 μg·g^−1^, the optimal adsorption conditions at pH = 7, and the adsorption performance after five adsorption–analysis cycles of this material was 93% of the initial adsorption capacity, and so on for the other materials.

The application of the aforementioned lithium ion-imprinted materials has been organized, analyzed, and summarized. Currently, lithium ion-imprinted materials exhibit high selectivity for lithium ions and can effectively extract lithium resources from environmental sources, thereby enhancing the efficiency of resource extraction. In recent studies, the maximum adsorption capacity reached 153.74 mg·g^−1^, positioning it at the forefront of research in this field. However, despite this achievement, the adsorption capacity still falls short of practical production requirements and therefore needs further improvement. Additionally, most materials are only suitable for neutral or weakly acidic/alkaline environments (with pH = 6–8 being predominant), indicating a limited applicability that requires enhancement. Moreover, these materials demonstrate a good reuse rate as their adsorption capacity after multiple cycles of adsorption–desorption remains approximately 90% of the initial value.

## 6. Summary and Prospects

In summary, compared with the previous conventional lithium ion-imprinted material preparation method, the new lithium ion-imprinted material preparation method combines the lithium ion-imprinting technology with other new technologies such as surface-imprinting technology, electrochemical technology, membrane separation technology, magnetic separation technology, etc., which solves the drawbacks of the conventional method to a certain extent, such as lower selectivity of the material to lithium ions and smaller adsorption capacity, and difficulty in material recycling. Although these problems are solved to a certain extent, it is still necessary to conduct some innovation and optimization for some acute problems.

First, improve the selectivity and adsorption capacity of the material for lithium ions. As there are not only lithium ions in the brine of the salt lake, but also a large number of alkali metal, alkaline earth metal ions and other interfering ions, it is particularly important to improve the selectivity and adsorption of materials for lithium ions. For the choice of complexing agent, we can choose crown ethers and their derivatives with strong effects on lithium ions, cuproaromatic hydrocarbons, and other high-efficiency complexing agents; in terms of carrier, we can choose graphene, carbon nanotubes, PVDF, and chitosan carriers that can provide more imprinted sites and further grafting and modification to improve the adsorption capacity of lithium ion-imprinted materials.

Secondly, regarding the recovery and reuse of imprinted materials, two primary challenges arise once lithium ions are eluted. Firstly, there is the issue of material recycling, followed by the value proposition of reusing the material post-recycling. Techniques such as the development of lithium ion-imprinted films, magnetic-imprinted materials, and imprinted adsorption columns facilitate swift and efficient material recycling. Maintaining high adsorption efficiency of lithium ion-imprinted materials after multiple uses necessitates addressing concerns like minimizing complexing agent shedding and loss. Achieving this requires employing stronger chemical bonds during material preparation and exploring more suitable complexing agents with greater chemical stability.

Thirdly, the environmental suitability of lithium ion-imprinted materials warrants further investigation. Presently, most materials exhibit optimal adsorption under relatively narrow conditions, typically within the neutral pH range (pH = 6–8). This limited applicability poses challenges for lithium extraction across diverse environmental conditions in China. Enhancing material adaptability to various environments is crucial to enable lithium extraction in a broader array of settings, necessitating improvements or optimizations in material properties.

Fourthly, the cost aspect presents a significant hurdle. Many experimental raw materials, including complexing agents and functional monomers, are prohibitively expensive. Additionally, the utilization of certain costly technologies further compounds the financial burden, potentially impeding research progress. Addressing this challenge entails exploring more cost-effective alternatives for functional monomers, cross-linking agents, initiators, carriers, solvents, and other components involved in material preparation, thus mitigating the financial barriers associated with research endeavors.

Finally, the practical application of lithium ion-imprinted materials remains largely confined to the laboratory research and development phase, with few transitioning to large-scale production. Urgent innovation is required to expedite this transition, enabling the adoption of new lithium ion-imprinted materials in actual production processes.

Lithium ion-imprinted materials have great potential and prospects in the field of lithium-ion batteries. As electric vehicles, portable electronic devices, and renewable energy sources rapidly evolve, the demand for high-performance and stable lithium-ion batteries is on the rise. Consequently, lithium ion-imprinted materials have emerged as a focal point of research due to their potential to enhance battery performance, safety, and environmental sustainability, while also reducing production costs. Overall, lithium ion-imprinted materials, as an important functional material, have a broad development prospect in lithium-ion batteries and related fields. Future research endeavors will prioritize enhancing material performance, cost reduction, safety improvements, and expanding application possibilities. These efforts aim to address the escalating demands for energy storage and environmental sustainability.

## Figures and Tables

**Table 1:** Calculation table of formulas related to performance evaluation of lithium ion-imprinted materials.

Project Name		Calculation Formula	Reference
Adsorption capacity *Q_t_* (mg·g^−1^)		$Q_{t}=\frac{V(C_{0}-C_{t})}{m}$	[31,32]
Isothermal adsorption model	Langmuir model	$Q_{e}=\frac{K_{L}Q_{m}C_{e}}{1+K_{L}C_{e}}$	[96,97]
	Freundlich model	$Q_{e}=K_{F}C_{e}^{\frac{1}{n}}$	[98]
	Tempkin model	$Q_{t}=A\ln k_{t}+\frac{1}{n}{\ln C}_{e}$	[99]
Kinetic model	Quasi-primary dynamics	$\frac{dq_{t}}{dt}=k_{1}\cdot (q_{e}-q_{t})$	[100]
	Quasi-secondary dynamics	$\frac{dq_{t}}{dt}=k_{2}\cdot {\left(q_{e}-q_{t}\right)}^{2}$	
Separation factor R_L_ value		$R_{L}=\frac{1}{1+C_{m}k_{L}}$	[101]
Selective evaluation equations	Distribution factor *K_d_*	$K_{d}=\frac{V(C_{0}-C_{e})}{mC_{e}}$	[31,102]
	Selection factor α	$\alpha =\frac{K_{d(Li^{+})}}{K_{d(Me)}}$	
	Relative selection factor α’	$\alpha^{\prime }=\frac{\alpha_{Me}^{Li^{+}}(IIP)}{\alpha_{Me}^{Li^{+}}(NIP)}$	

**Table 2:** A summary of the preparation systems and adsorption capacities of different lithium ion-imprinted materials.

The Name of the Material	Type of Material	Synthesis Method	Template Ions	Complexing Agent	Functional Monomers	Cross-linker	Initiator	Carrier	Solvent Adsorption	Capacity	References
Li/Rb-IHPS	Adsorption columns	Precipitation polymerization method	LiCl·H_2_O	12C4	MAA	EGDMA	AIBN	HPS	CH_3_CN	2321.6 μg·g^−1^	[30]
Li^+^-IIP	Imprinted adsorption columns	Surface-imprinted polymerization method	LiNO_3_	DB14C4	MAA	EGDMA	AIBN	MWCNT	DMSO	32.23 μmol·g^−1^	[84]
Fe_3_O_4_@SiO_2_@IIP	Magnetic imprinted material	Surface-imprinted polymerization method	LiCl·H_2_O	2M12C4	TEOS	EGDMA	AIBN	MH-Fe_3_O_4_@SiO_2_	CH_3_OH and DMF	0.586 mmol·g^−1^	[38]
Fe_3_O_4_@C@IIP	Magnetic imprinted material	Surface-imprinted polymerization method	LiClO_4_	B12C4	NIPAM	EGDMA	AIBN	Fe_3_O_4_@C	CH_3_CN	23.46 mg·g^−1^	[31]
IIP-GO/Fe_3_O_4_@C	Magnetic imprinted material	Surface-imprinted polymerization method	LiClO_4_	B12C4	MAA	EGDMA	AIBN	GO/Fe_3_O_4_@C	DMF	31.24 mg·L^−1^	[32]
Li^+^-IIP-Fe_3_O_4_@C	Magnetic imprinted material	Surface-imprinted polymerization method	LiClO_4_	2M12C4	MAA	EGDMA	AIBN	GO/Fe_3_O_4_@C	DMF	31.24 mg·L^−1^	[104]
IIMMs	Imprinted membrane material	Surface-imprinted polymerization method	LiCl	2M12C4	DA	EGDMA	AIBN	PVDF	CH_3_OH	27.1 mg·g^−1^	[105]
IINcMs	Imprinted membrane material	Surface-imprinted polymerization method	LiCl	2M12C4	PDA	EGDMA	AIBN	MPTS-Ag/PDA/PVDF	CH_3_OH	25.58 mg·g^−1^	[40]
LIIP@N-CMS/GA	Imprinted membrane material	Electrochemical	LiClO_4_	B12C4	GO	PPy	/	N-CMS/GA	KCl	59.58 mg·g^−1^	[39]
Imprinted membrane material	Imprinted membrane material	Electrochemical	LiCl	2M12C4	/	Py	/	/	KCl	16.4 mg·g^−1^	[42]
LIHMs	Imprinted membrane material	Hydrolysis polymerization method	LiCl	12C4E	APTES	TEOS	/	PVDF	C_2_H_5_OH	132 mg·g^−1^	[44]
LIHMs	Imprinted membrane material	Hydrolysis polymerization method	LiCl	12C4E	VTES	TEOS	/	pDA@GO/PVDF	C_2_H_5_OH	27.10 mg·g^−1^	[41]
IDGAs	Imprinted aerogel material	Precipitation polymerization method	LiClO_4_	2M12C4	MAA	DGDMA	AIBN	DGA	C_2_H_5_OH	11.50 mg·g^−1^	[45]

**Table 3:** Adsorption capacity range, optimum pH and regeneration performance parameters of Li-IIPs.

The Name of the Material	Adsorption Capacity Range	Optimal pH	Number of Adsorption–Desorption	Remaining Adsorption Capacity	References
Li/Rb-IHPS	0–200 μg·g^−1^	7	5	93%	[30]
Li^+^-IIP	0–1400 μmol·g^−1^	6	10	89.7%	[84]
N@Li^+^-IPDI	0–15 mg·g^−1^	6	5	93.42%	[43]
Fe_3_O_4_@SiO_2_@IIP	0–1 mmol·g^−1^	6	5	92.4%	[38]
Li^+^-IIP	0–30 mg·g^−1^	/	5	91.47%	[31]
IIP-GO/Fe_3_O_4_@C	0–30 mg·g^−1^	6	6	91%	[32]
Li^+^-IIP-Fe_3_O_4_@C	0–15 mg·g^−1^	/	6	92%	[104]
IIMMs	20–200 mg·g^−1^	9	6	90.91%	[105]
IINcMs	2–50 mg·g^−1^	/	10	92.10%	[40]
LIIP@N-CMS/GA	0–60 mg·g^−1^	9	10	91.70%	[39]
Li^+^-IIM	5–50 mg·g^−1^	7	5	95.88%	[42]
LIIMs	0–200 mg·g^−1^	/	6	97%	[44]
LIHMs	20–200 mg·g^−1^	6	6	91.80%	[41]
LIIP@N-CMS/GA	10–200 mg·g^−1^	9	10	91.7%	[39]
LIIMs	30–700 mg·g^−1^	/	6	97%	[44]
IDGAs	0–15 mg·g^−1^	/	4	88.50%	[45]
polyHIPE	0–5 mg·g^−1^	6	5	96.4%	[46]
IIP@SG/GO	0–2 mg·g^−1^	8	5	89.09%	[47]
